# Design, Synthesis, and Biological Evaluation of Novel Nitrogen Heterocycle-Containing Ursolic Acid Analogs as Antitumor Agents

**DOI:** 10.3390/molecules24050877

**Published:** 2019-03-01

**Authors:** Wenzhi Wang, Lei Lei, Zhi Liu, Hongbo Wang, Qingguo Meng

**Affiliations:** School of Pharmacy, Key Laboratory of Molecular Pharmacology and Drug Evaluation (Yantai University), Ministry of Education, Collaborative Innovation Center of Advanced Drug Delivery System and Biotech Drugs in Universities of Shandong, Yantai University, Yantai 264005, China; wxwnx123@163.com (W.W.); 18363855773@163.com (L.L.); zhi_liu0812@163.com (Z.L.)

**Keywords:** ursolic acid analogues, synthesis, nitrogen heterocycles, apoptosis, antitumor activity

## Abstract

Nineteen ursolic acid analogues were designed, synthesized, and evaluated for their antiproliferative activity against the Hela and MKN45 cell lines. Some compounds containing a piperazine moiety displayed moderate to high levels of antitumor activities against the tested cancer cell lines. The most potent compound shares the IC_50_ value of 2.1 µM and 2.6 µM for the Hela and MKN45 cell lines, respectively. Further mechanism studies and in vivo antitumor studies have shown that it decreased the apoptosis regulator (BCL2/BAX) ratio, disrupted mitochondrial potential and induced apoptosis, and suppressed the growth of Hela xenografts in nude mice.

## 1. Introduction

Ursolic acid (3*β*-hydroxy-12-urs-12-en-28-oic acid, UA) is a pentacyclic triterpenoid that occurs naturally in many vegetables, fruits, and Chinese medicinal herbs [1,2]. UA and its derivatives possess extensive pharmacological effects, including antiviral [3], antibacterial [4,5], anticancer [6,7], antiosteoporosis [8], antidiabetic [9,10], anti-inflammatory, and hepatoprotective activity [11,12]. In recent years, increasing attention has been focused on the antitumor activity and the possible mechanisms of UA and its derivatives. It has been reported that they exert antitumor activity by a number of different mechanisms. These include inducing apoptosis and differentiation, inhibition of proliferation and angiogenesis, promotion of chemosensitization, and induction of cell cycle arrest [13]. Intense efforts have been made to endow UA with higher water solubility and to achieve an improved antineoplastic effect [2,14,15,16].

Nitrogen heterocycles are significant structural components of most new synthetic molecules that are endowed with significant biological activity [17,18] and pharmaceuticals. More than 59% of unique small molecule drugs that are approved by the FDA contain nitrogen heterocycles [19]. Studies showed that 3-oxo-UA-triazolyl derivatives with *o*-bromo, *o*-chloro, or *o*-methoxy substitution on the aromatic ring more potently inhibit the growth of MCF-7 and THP-1 cancer cell lines [20]. UA analogues containing an acyl piperazine moiety at C-28 exhibit high inhibitory activity against MGC-803 and Bcap-37 cancer cell lines and promote apoptosis [21]. In addition, some UA derivatives containing quinoline displayed potent antitumor activity against MDA-MB-231, Hela, and SMMC-7721 cell lines [22].

**Scheme 1:**
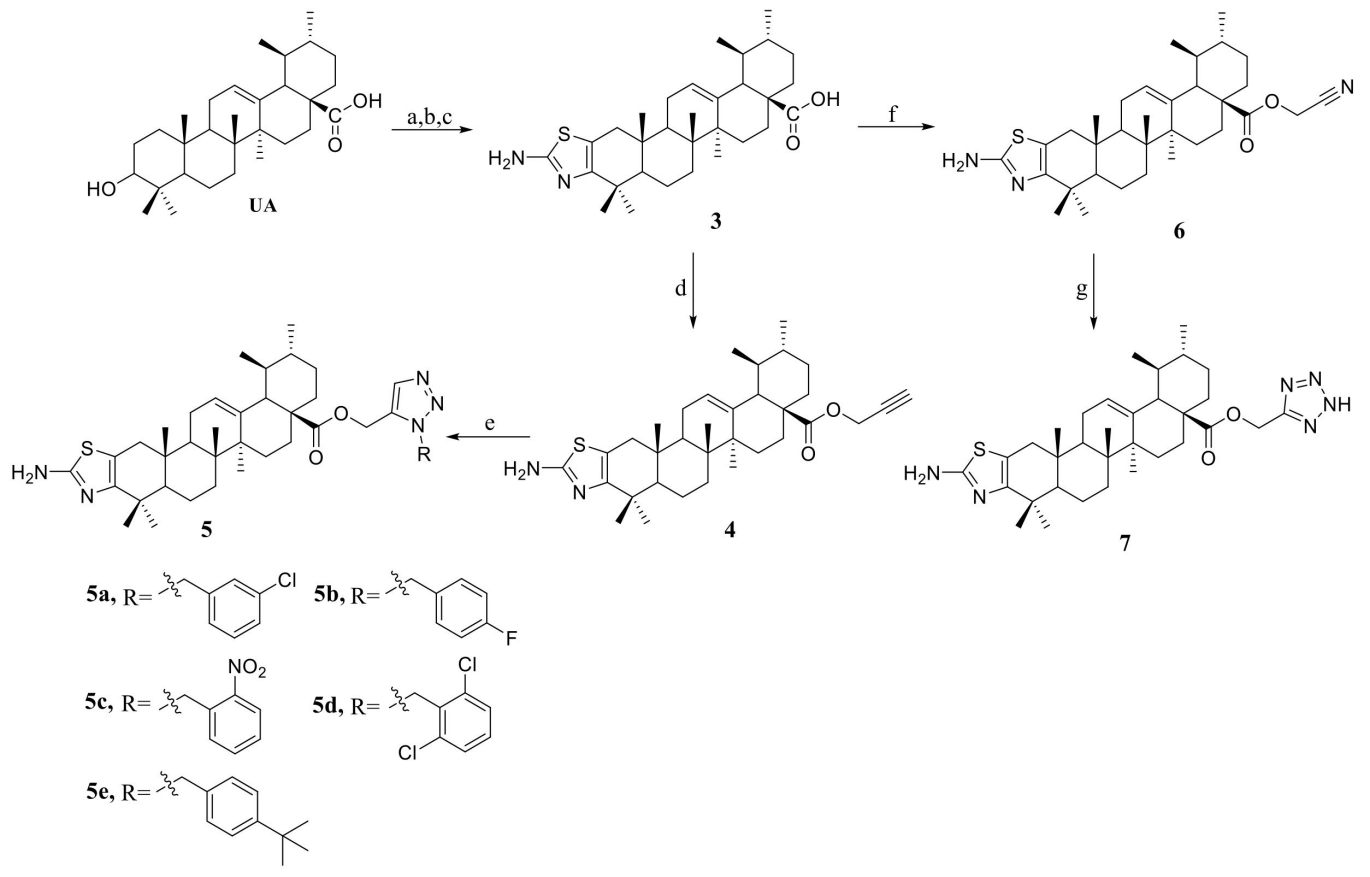
Synthesis of compounds **3**–**7.** Reagents and conditions: (**a**) PCC, CHCl_3_, rt, 6 h (**b**) pyridinium tribromide, HOAc, rt, 6 h (**c**) thiourea, 80 °C, 10 h (**d**) 3-bromo-propyne, K_2_CO_3_, *N*,*N*-dimethylformamide (DMF), 60 °C 1 h (**e**) N_3_-R, CuSO_4_·5H_2_O, sodium ascorbate, EtOH, H_2_O, rt, 4 h (**f**) chloroacetonitrile, K_2_CO_3_, DMF, 60 °C 1 h (**g**) NaN_3_, ZnCl_2_, isopropanol/H_2_O, 85 °C, 6 h.

**Scheme 2:**
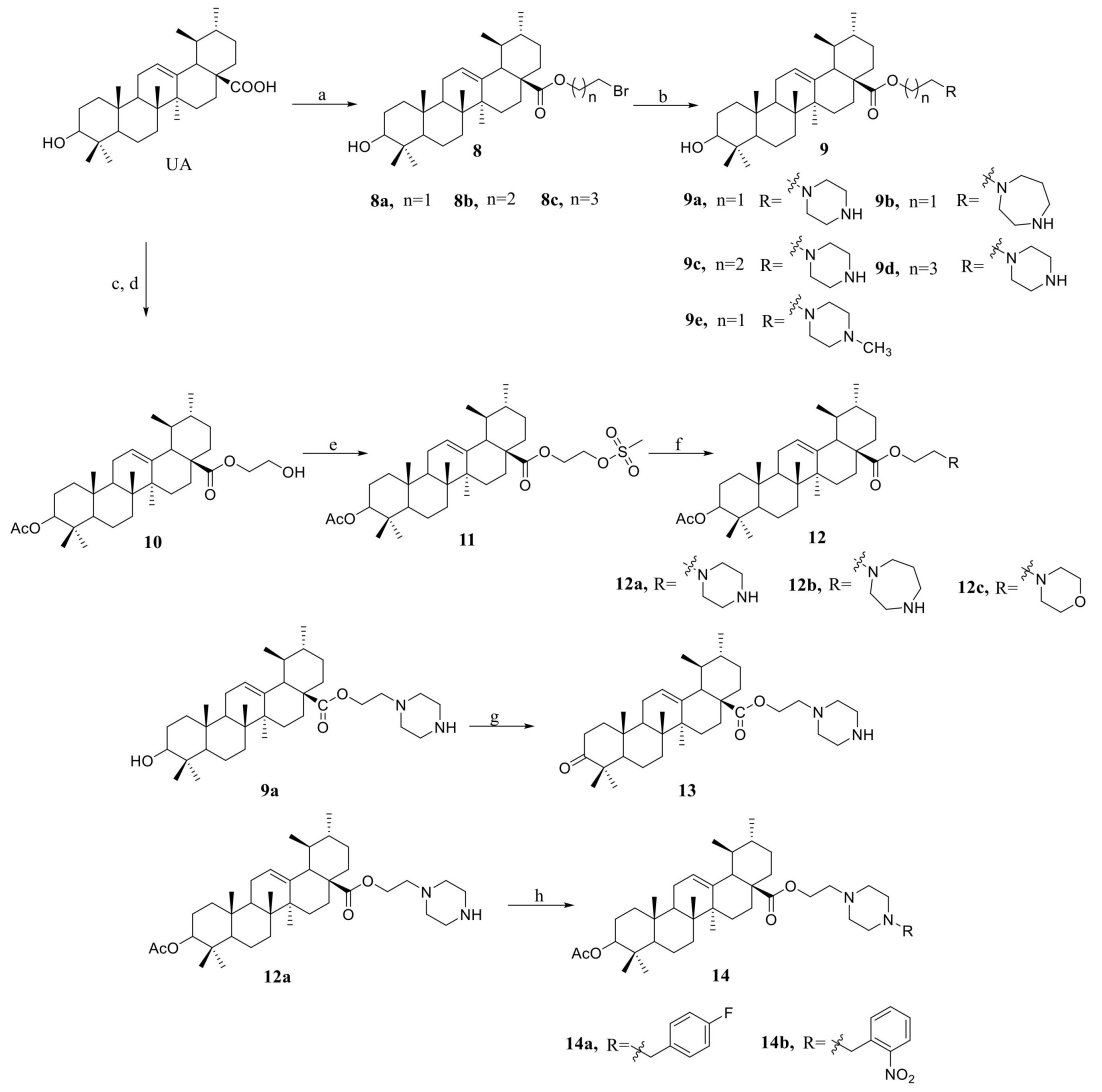
Synthetic pathways of compounds **8**–**14**. Reagents and conditions: (**a**) 1,2-dibromoethane or 1,3-dibromopropane or 1,4-dibromobutane, K_2_CO_3_, DMF, 60 °C, 1 h (**b**) piperazine or homopiperazine or 1-methylpiperazine, K_2_CO_3_, DMF, 80 °C, 30–60 min (**c**) Ac_2_O, pyridine, 0 °C~r.t, 15 h (**d**) 2-chloroethanol, K_2_CO_3_, DMF 60 °C, 3 h (**e**) MsCl, pyridine, 10 °C~r.t, 3 h (**f**) piperazine or homopiperazine or morpholine, K_2_CO_3_, DMF, 60 °C, 30 min (**g**) PCC, CHCl_3_, 0 °C, 10 h (**h**) 4-fluorobenzyl bromide or 2-nitrobenzyl bromide, K_2_CO_3_, DMF, 60 °C 1 h.

**Scheme 3:**
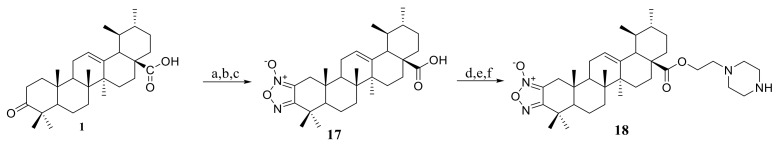
Synthesis of **17** and **18**. Reagents and conditions: (**a**) isoamyl nitrite, *t*-BuOK, *t*-BuOH, r.t, 2.5 h (**b**) hydroxylamine hydrochloride, pyridine, 110 °C, 2 h (**c**) EtOH, NaOH, NaOCl, rt, 2 min (**d**) 2-chloroethanol, K_2_CO_3_, DMF, 60 °C, 3 h (**e**) MsCl, pyridine, 10 °C~r.t, 3 h (**f**) piperazine, K_2_CO_3_, DMF, 60 °C, 30 min.

Using a drug discovery strategy that is based on nitrogen heterocycles, nineteen UA analogues containing nitrogen heterocycles were designed and synthesized (Scheme 1, Scheme 2 and Scheme 3). Their antitumor activities were evaluated and the possible mechanism of Hela cell growth inhibition was also investigated.

## 2. Results and Discussion

### 2.1. Chemistry

The compounds were synthesized, as outlined in the Scheme 1, Scheme 2 and Scheme 3. The initial key intermediate **3** was synthesized according to published protocols [23]. As shown in Scheme 1, compound **3** was treated with 3-bromo-1-propyne or chloroacetonitrile in *N*,*N*-dimethylformamide (DMF), followed by a click reaction with corresponding azides to introduce a triazole or tetrazole moiety (**5a**–**e** and **7**). Compounds **8a**–**c** were synthesized from UA and dibromoalkane, and then treated with piperazine, homopiperazine, or 1-methylpiperazine to gain derivatives **9a**–**e** with a moderate yield (38–43%). Among them, compounds **9a** and **9c** have been reported as antitumor agents by M.C. Liu and X. Li, respectively [21,24]. UA was subjected to acetylation and reacted with 2-chloroethanol to obtain compound **10**, followed by sulfonylation to generate derivative **11**. Subsequently, compound **11** was treated with piperazine, homopiperazine, or morpholine to afford derivatives **12a**–**c** with a yield of 71–75%. Compound **13** was synthesized from compound **9a** by oxidation with PCC in chloroform. Compound **12a** was treated with 4-fluorobenzyl or 2-nitrobenzyl bromide to generate derivatives **14a**–**b** (Scheme 2).

Compound **17**, bearing a furazan moiety at C-2 and C-3 of UA, was synthesized according to the published method [25]. It was further spliced with an ethyl-piperazine to yield compound **18** (Scheme 3). All of the target compounds were purified by silica gel column chromatography. ^1^H-NMR, ^13^C-NMR and HR-MS spectra of compounds are shown in Supplementary Materials.

### 2.2. Biological Evaluation

The target compounds were screened for antiproliferative activity against Hela and MKN45 cancer cell lines using an MTT assay. Cisplatin and UA were used as positive controls and the preliminary results are presented in Table 1. Compounds **5a**–**e** and **7** shared low to moderate antiproliferative activity (inhibition rates from 1–45%) against Hela and MKN45 cancer cells at the tested concentration (10 µM). Compounds **9a**, **9c**, **12a**, **12b**, **13**, **14a,** and **18** displayed much higher activity than UA and possessed IC_50_ values that were lower than 10 µM. Nevertheless, derivatives **9e**, **12c, 14b,** and **17** showed moderate to low activity against the two cancer cell lines.

The structure activity relationship studies revealed that the introduction of thiazole on A ring and triazole or tetrazole moiety on C-28 have little help to improve antitumor activities. The introduction of piperazine or homopiperazine can significantly improve the antitumor activity. The length of the carbon chains between the carboxyl and piperazine moiety of compounds **9a**, **9c,** and **9d** could have affected the antiproliferative activity. The two- or three-carbon chain linker appears to the optimum, **9a** and **9c** with a straight two- and three -carbon chain showed good antitumor activities against the two cell lines. Compounds **12a** and **12b** possess more potent anticancer activity when compared with **9a** and **9b**, which may be attributed to the introduced acetyl group. **14a** showed much higher antiproliferative activity than both UA and cisplatin with IC_50_ values of 2.1 µM and 2.6 µM for Hela and MKN45, respectively. Compound **14b** displayed weaker antiproliferative activity as compared with UA. These results demonstrated that 4-fluorobenzyl and piperazine moieties are the crucial anticancer functional groups of these compounds.

**Figure 1:**
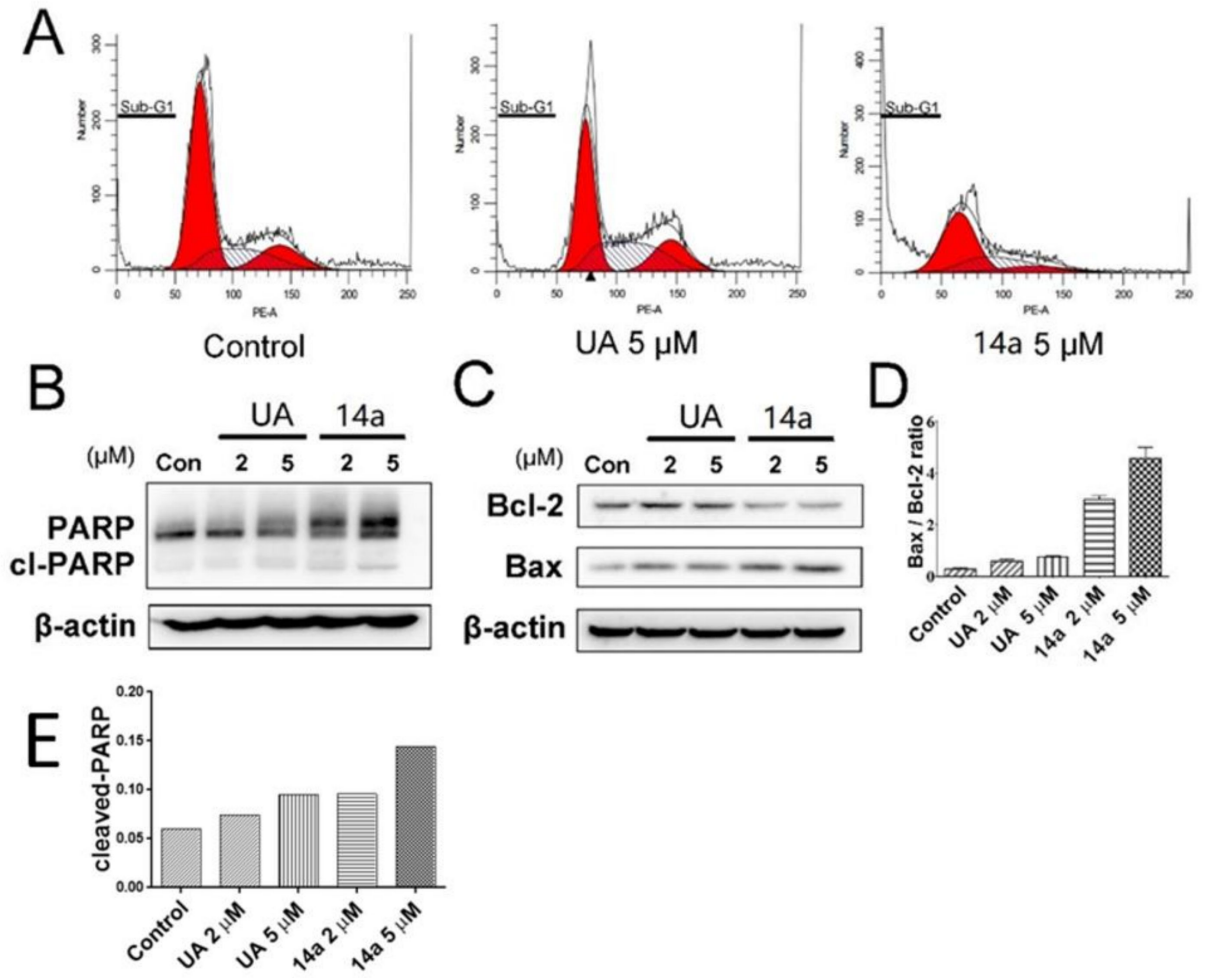
Apoptotic profile of compound **14a** in Hela cells. (**A**) DNA cell cycle analyses of Hela cells exposed to **14a**. The cells were treated with indicated concentrations of **14a** for 24 h and stained with propidium iodide (PI, 50 µg/mL) to determine DNA fluorescence and cell cycle phase distribution. (**B**) and (**C**) Effect of **14a** on the expression of key apoptotic-related proteins. Cells (2 × 10^5^) were treated with 2 µM or 5 µM of **14a** for 24 h before cell lysates were prepared and analyzed. β-actin was used as an internal control. (**D**) The relative density of BCL2 and BAX was measured as arbitrary units by ImageJ software and was used to calculate the BAX/BCL2 ratio. (**E**) The relative density of cleaved poly-ADP ribose polymerase also was measured as arbitrary units by ImageJ software. Data are representative of three experiments.

Since the inhibition of apoptosis is one of hallmarks of cancer, the effects of compound **14a** on cell cycle progression were examined by the propidium iodide staining method. The results showed that compound **14a**, but not UA, at a concentration of 5 µM increased the proportion of cells in the sub-G1 phase (Figure 1A). This indicated that **14a** induced cell death. Consistent with this, western blotting showed that **14a** increased the abundance of cleaved poly-ADP ribose polymerase (PARP), which is a biomarker of apoptosis (Figure 1B). The disruption of the mitochondrial membrane potential, regulated by BCL2 family of proteins, such as BCL2 and BAX, is a mechanism that triggers apoptosis following treatment by anticancer drugs. Our results showed that treatment with **14a** decreased the expression of BCL2, one of the main mitochondrial-associated antiapoptotic proteins. **14a** also increased the expression of BAX, a key mitochondrial-associated apoptotic protein, in a dose-dependent manner (Figure 1C). Therefore, our results suggest that compound **14a** induces apoptosis via the intrinsic mitochondrial pathway.

**Figure 2:**
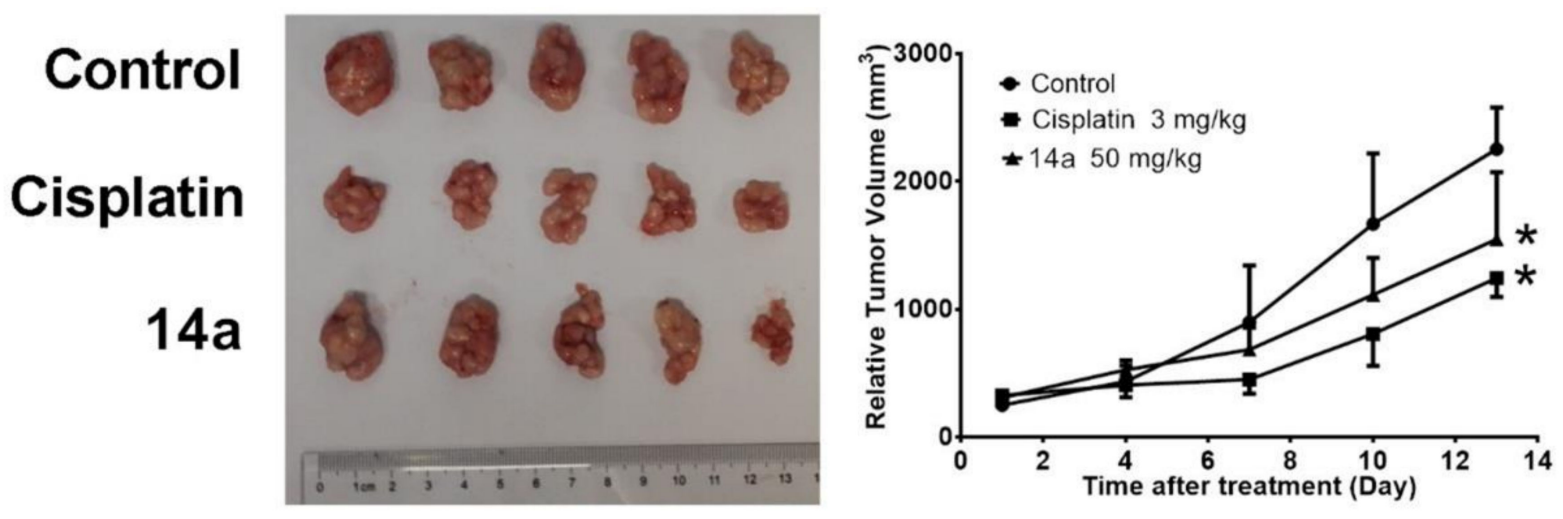
Effect of **14a** on the growth of Hela in nude mice. Tumor diameter was serially measured with a vernier caliper to calculate the relative tumor volume. Each experiment was conducted using 5 mice per group. Vehicle (•); 50 mg/kg **14a** (▲); and, 3 mg/kg cisplatin (■). The statistical difference between the drug-treated group and the vehicle was determined by one-way ANOVA (* *p* < 0.05).

A xenograft model in nude mice was established and used to evaluate the in vivo antitumor activity of **14a**. The mice were treated with vehicle, cisplatin (3 mg/kg, i.p.) and 50 mg/kg of **14a**. Our results showed that **14a** could significantly suppress growth of Hela xenograft tumors and had a similar effect to cisplatin treatment (Figure 2 and Table 2).

## 3. Materials and Methods

### 3.1. Materials

Ursolic acid (>95%) was purchased from Shaanxi Pioneer Biotech Co., Ltd (Hanzhong, China). Reagents and solvents were either analytical or chemical purity. The silica gel GF_254_ plates and silica gel (200 to 300 mesh, Qingdao, Shandong, China) were used. The melting points were determined using an XT3A micro-melting point apparatus and are uncorrected (Beijing Keyi Company, Beijing, China). ^1^H NMR and ^13^C NMR were recorded on the Avance-400 spectrometer (Bruker, Ettlingen, Germany). High resolution mass spectra (HR-MS) were obtained on an AutoSpec Ultima-Tof spectrometer with an electrospray ionization (ESI) source (Micromass, Manchester, UK).

### 3.2. Chemistry

#### 3.2.1. Preparation of Compounds **3**, **4** and **6**

Compound **3** was synthesized by the literature method with slight modifications [21]. The solution of **3** (500 mg, 0.98 mmol), K_2_CO_3_ (135 mg, 0.98 mmol), and 3-bromopropyne (0.11 mL, 1.28 mmol) or chloroacetonitrile (0.08 mL, 1.26 mmol) in DMF (20 mL) was stirred at 60 °C for 1 h. After cooled to room temperature, the mixture was poured into ice-cold water and filtered. The solid was dissolved with ethyl acetate (100 mL) and washed with water (50 mL) and brine (50 mL × 2), dried over Na_2_SO_4_ anhydrous, filtered, and concentrated to obtain **4** and **6**, as yellowish-white powder, in 92% and 95% yield, respectively.

#### 3.2.2. General Procedure for the Synthesis of Compounds **5a**–**e**

To a solution of compound **4** (200 mg, 0.36 mmol) in anhydrous alcohol (5 mL), CuSO_4_·5H_2_O (37 mg, 0.15 mmol), sodium ascorbate (18 mg, 0.09 mmol), and corresponding benzyl azide (0.6 mmol) were added. The mixture was stirred at room temperature for 4 h and then concentrated. The residue was dissolved with ethyl acetate (80 mL), washed with water (40 mL) and brine (40 mL × 2), and then dried over Na_2_SO_4_ anhydrous. After filtration, concentration and purification by silica gel column chromatography with cyclohexane/methanol (3:1), compounds **5a**–**e** were afforded.

*[1-(3-chlorobenzyl)-1H-1,2,3-triazol-5-yl]methyl-urs-12-en-[(3,2-d)thiazol-2-amino]-28-oate (**5a**)*. 26%, off-white powder. mp: 120.1–122.5 °C. ^1^H NMR (400 MHz, CDCl_3_): *δ* 7.51 (1H, s), 7.36–7.27 (2H, m), 7.23 (1H, s), 7.14 (1H, d, *J* = 7.0 Hz), 5.53–5.42 (2H, m), 5.22 (1H, s), 5.19–5.09 (2H, m), 2.47 (1H, d, *J* = 15.4 Hz), 2.21 (1H, d, *J* = 11.5 Hz), 2.16 (1H, d, *J* = 15.6 Hz), 1.25 (6H, s), 1.20 (3H, s), 1.12 (3H, s), 1.06 (3H, s), 0.92 (6H, s); ^13^C NMR (100 MHz, CDCl_3_): *δ* 177.64, 164.78, 152.08, 143.92, 138.17, 136.52, 135.20, 130.54, 129.20, 128.20, 126.21, 125.57, 124.04, 116.04, 57.45, 53.54, 53.04, 52.68, 48.32, 46.14, 42.34, 39.68, 39.26, 38.94, 38.89, 38.33, 36.95, 36.63, 32.50, 30.75, 30.49, 28.09, 24.30, 23.43, 23.31, 22.29, 21.25, 19.66, 17.08, 16.79, 15.72. HR-MS *m/z* calcd. for C_41_H_55_ClN_5_O_2_S [M + H]^+^ 716.37595, found 716.37531.

*[1-(4-fluorobenzyl)-1H-1,2,3-triazol-5-yl]methyl-urs-12-en-[(3,2-d)thiazol-2-amino]-28-oate (**5b**)*. 26%, off-white powder. mp: 115.0–116.9 °C; ^1^H NMR (400 MHz, CDCl_3_): *δ* 7.47 (1H, s), 7.30–7.22 (2H, m), 7.05 (2H, t, *J* = 8.5 Hz), 5.46 (2H, s), 5.21 (1H, s), 5.18–5.06 (2H, m), 2.47 (1H, d, *J* = 15.4 Hz), 2.20 (1H, d, *J* = 12.7 Hz), 2.16 (1H, s), 1.24 (3H, s), 1.20 (3H, s), 1.11 (3H, s), 1.06 (3H, s), 0.92 (6H, s), 0.83 (3H, d, *J* = 6.4 Hz), 0.59 (3H, s); ^13^C NMR (100 MHz, CDCl_3_): *δ* 177.64, 164.75, 163.01 (d, *J* = 248.4 Hz), 152.13, 143.81, 138.17, 130.45 (d, *J* = 3.3 Hz), 130.13 (d, *J* = 8.4 Hz), 125.56, 123.84, 116.30 (d, *J* = 21.8 Hz), 57.47, 53.53, 53.05, 52.68, 48.32, 46.14, 42.34, 39.68, 39.26, 38.94, 38.91, 38.34, 36.96, 36.65, 32.51, 31.08, 30.76, 30.49, 28.08, 24.30, 23.44, 23.34, 22.26, 21.26, 19.64, 17.09, 16.77, 15.72. HR-MS *m/z* calcd. for C_41_H_55_FN_5_O_2_S [M + H]^+^ 700.40550, found 700.40482.

*[1-(2-nitrobenzyl)-1H-1,2,3-triazol-5-yl]methyl-urs-12-en-[(3,2-d)thiazol-2-amino]-28-oate (**5c**)*. 28%, off-white powder. mp: 130.3-132.5 °C; ^1^H NMR (400 MHz, CDCl_3_): *δ* 8.15 (1H, d, *J* = 8.1 Hz), 7.71 (1H, s), 7.53–7.60 (2H, m), 7.09 (1H, d, *J* = 7.7 Hz), 5.91 (2H, s), 5.25 (1H, s), 5.23–5.13 (2H, m), 2.48 (1H, d, *J* = 15.3 Hz), 2.23 (1H, d, *J* = 11.4 Hz), 2.17 (1H, d, *J* = 14.8 Hz), 1.20 (3H, s), 1.12 (3H, s), 1.07 (3H, s), 0.93 (6H, d, *J* = 1.0 Hz), 0.65 (3H, s); ^13^C NMR (100 MHz, CDCl_3_): *δ* 177.55, 164.60, 147.58, 143.85, 138.12, 134.44, 130.57, 129.88, 125.64, 125.54, 124.99, 116.11, 57.45, ^53^.06, 52.66, 50.92, 48.32, 46.13, 42.32, 39.68, 39.24, 38.94, 38.89, 38.33, 36.95, 36.65, 32.49, 30.75, 30.53, 28.08, 24.28, 23.45, 23.34, 22.30, 21.24, 19.66, 17.07, 16.84, 15.70. HR-MS *m/z* calcd. for C_41_H_55_N_6_O_4_S [M + H]^+^ 727.40000, found 727.39938.

*[1-(2,6-dichlorobenzyl)-1H-1,2,3-triazol-5-yl]methyl-urs-12-en-[(3,2-d)thiazol-2-amino]-28-oate (**5d**)*. 260%, off-white powder. mp: 136.0–138.8 °C; ^1^H NMR (400 MHz, CDCl_3_): *δ* 7.50 (1H, s), 7.40 (2H, d, *J* = 7.9 Hz), 7.33–7.27 (1H, m), 5.83 (2H, s), 5.22 (1H, s), 5.17–5.07 (2H, m), 4.92 (2H, s), 2.48 (1H, d, *J* = 15.4 Hz), 2.20 (1H, d, *J* = 11.7 Hz), 2.19–2.14 (2H, m), 1.20 (3H, s), 1.12 (3H, s), 1.06 (3H, s), 0.93 (6H, s), 0.83 (3H, d, *J* = 6.4 Hz), 0.58 (3H, s); ^13^C NMR (100 MHz, CDCl_3_): *δ* 177.58, 164.64, 152.24, 143.14, 138.19, 136.95, 131.26, 130.05, 129.03, 125.55, 123.75, 116.09, 57.43, 53.03, 52.67, 49.15, 48.29, 46.13, 42.30, 39.66, 39.24, 38.96, 38.90, 38.33, 36.96, 36.65, 32.47, 30.76, 30.50, 28.06, 24.25, 23.45, 23.36, 22.28, 21.25, 19.68, 17.08, 16.67, 15.73. HR-MS *m/z* calcd. for C_41_H_54_Cl_2_N_5_O_2_S [M + H]^+^ 750.33698, found 750.33651.

*{1-[4-(tert-butyl)benzyl]-1H-1,2,3-triazol-5-yl}methyl-urs-12-en-[(3,2-d)thiazol-2-amino]-28-oate (**5e**)*. 26%, off-white powder. mp: 149.1–151.3 °C; ^1^H NMR (400 MHz, CDCl_3_): *δ* 7.46 (1H, s), 7.38 (2H, d, *J* = 8.3 Hz), 7.18 (2H, d, *J* = 8.3 Hz), 5.53–5.39 (2H, m), 5.22 (1H, s), 5.09–5.17 (2H, m), 4.86 (2H, s), 2.50 (1H, d, *J* = 15.4 Hz), 1.30 (9H, s), 1.21 (3H, s), 1.12 (3H, s), 1.07 (3H, s), 0.97 (3H, s), 0.92 (3H, s), 0.83 (3H, d, *J* = 6.4 Hz), 0.66 (3H, s); ^13^C NMR (100 MHz, CDCl_3_): *δ* 177.59, 164.59, 152.19, 152.10, 143.58, 138.17, 131.50, 128.04, 126.17, 125.59, 123.89, 116.19, 57.50, 54.00, 53.06, 52.70, 48.29, 46.18, 42.35, 39.71, 39.26, 39.00, 38.91, 38.37, 36.97, 36.61, 32.53, 31.39, 30.77, 30.53, 28.10, 24.29, 23.46, 23.36, 22.32, 21.26, 19.68, 17.09, 16.85, 15.77. HR-MS *m/z* calcd. for C_45_H_64_N_5_O_2_S [M + H]^+^ 738.47752, found 738.48079.

*(2H-tetrazol-5-yl) methyl-urs-12-en-[(3,2-d)thiazol-2-amino]-28-oate (**7**)*. The solution of **6** (200 mg, 0.36 mmol), sodium azide (48 mg, 0.74 mmol) and ZnCl_2_ (196 mg, 1.4 mmol) in isopropanol/water (3:1, 8 mL) was added and stirred at 85 °C for 6 h. After concentration, the residue was dissolved with *n*-butanol (60 mL) and washed with water (30 mL) and brine (30 mL × 2), dried over Na_2_SO_4_ anhydrous, filtered, evaporated and purified by silica gel column chromatography with chloroform/methanol (10:1) to give **7**. 31%, white powder. mp: 256.0–258.0 °C; ^1^H NMR (400 MHz, DMSO-*d*_6_): *δ* 6.70 (2H, s), 5.28 (2H, s), 5.15 (1H, s), 2.40 (1H, d, *J* = 15.4 Hz), 2.16 (1H, d, *J* = 11.3 Hz), 2.08 (1H, d, *J* = 15.5 Hz), 1.10 (3H, s), 1.04 (3H, s), 1.02 (3H, s), 0.91 (3H, s), 0.86 (3H, d, *J* = 7.7 Hz), 0.81 (2H, d, *J* = 6.3 Hz), 0.51 (3H, s); ^13^C NMR (100 MHz, DMSO*-d*_6_): *δ* 175.83, 165.29, 153.23, 150.88, 137.41, 125.21, 112.23, 54.61, 52.58, 52.04, 47.63, 45.35, 39.00, 38.38, 38.36, 38.21, 37.78, 36.43, 35.98, 32.02, 30.39, 30.01, 27.45, 23.73, 23.10, 22.76, 22.12, 20.95, 19.14, 16.92, 16.30, 15.41. HR-MS *m/z* calcd. for C_33_H_49_N_6_O_2_S [M + H]^+^ 593.36322, found 593.36123.

#### 3.2.3. General Procedure for Compounds **8a**–**c**

To a solution of UA (1.10 g, 2.41 mmol), K_2_CO_3_ (0.49 g, 3.55 mmol) and corresponding dibromoalkane (8.0 mmol) in 15 mL DMF, the mixture was stirred at 60 °C for 1 h. This was followed by the similar post-reaction treatment of compounds **4** or **5**, purified by silica gel column chromatography with petroleum ether/ethyl acetate (5:1) to afford derivatives **8a**–**c**, as yellowish-white powder, 61–63% yield.

#### 3.2.4. General Procedure for Compounds **9a**–**e**

K_2_CO_3_ (1 equiv) and the corresponding nitric heterocyclic compounds (2–6 equiv) were added to the solution of **8a**, **8b,** or **8c** (1 equiv) in DMF. The mixture was stirred at 80 °C for 30–60 min and then poured into ice-cold water, stirred, and filtered. The crude product was dissolved in ethyl acetate, washed with water and brine, dried over Na_2_SO_4_ anhydrous, filtered, evaporated, and purified by silica gel column chromatography to yield **9a**–**e**.

*[2-(Piperazin-1-yl)ethyl] 3-hydroxy-urs-12-en-28-oate(**9a**)*. 40% (purified by silica gel column chromatography with dichloromethane/methanol, 10:1), white solid. mp: 180.0–182.0 °C; ^1^H NMR (400 MHz, CDCl_3_): *δ* 5.23 (1H, s), 4.19–4.07 (2H, m), 3.22 (1H, dd, *J* = 10.8, 4.6 Hz), 3.14 (4H, s), 2.75 (4H, s), 2.65 (2H, t, *J* = 5.6 Hz), 2.20 (1H, d, *J* = 11.3 Hz), 1.08 (3H, s), 0.99 (3H, s), 0.95 (3H, d, *J* = 5.5 Hz), 0.92 (3H, s), 0.86 (3H, d, *J* = 6.3 Hz), 0.79 (3H, s), 0.74 (3H, s); ^13^C NMR (100 MHz, CDCl_3_): *δ* 176.89, 137.61, 125.09, 78.47, 61.24, 56.67, 54.70, 54.06, 52.34, 47.48, 47.03, 45.52, 41.52, 39.04, 38.56, 38.37, 38.25, 38.10, 36.46, 36.18, 32.53, 30.17, 27.65, 27.47, 26.73, 23.71, 23.05, 22.78, 20.70, 17.81, 16.65, 16.54, 15.15, 14.97. HR-MS *m/z* calcd. for C_36_H_61_N_2_O_3_ [M + H]^+^ 569.46767, found 569.46555.

*[2-(homopiperazine-1-yl)ethyl] 3-hydroxy-urs-12-en-28-oate(**9b**)*. 39% (purified by silica gel column chromatography with dichloromethane/methanol, 10:1), white solid. mp: 189.2–192.0 °C; ^1^H NMR (400 MHz, CDCl_3_): *δ* 5.22 (1H, s), 4.14–4.02 (2H, m), 3.27–2.78 (9H, m), 2.18 (1H, d), 1.07 (3H, s), 0.98 (3H, s), 0.93 (3H, d, *J* = 5.7 Hz), 0.91 (3H, s), 0.84 (3H, d, *J* = 6.4 Hz), 0.77 (3H, s), 0.73 (3H, s); ^13^C NMR (100 MHz, CDCl_3_): *δ* 177.51, 138.17, 125.72, 79.09, 61.92, 56.43, 55.29, 54.00, 53.01, 51.03, 48.15, 47.61, 46.97, 44.67, 42.15, 39.63, 39.14, 38.96, 38.84, 38.70, 37.06, 36.86, 33.15, 30.72, 28.26, 28.09, 27.31, 25.36, 24.35, 23.62, 23.40, 21.26, 18.41, 17.27, 17.13, 15.77, 15.60. HR-MS *m/z* calcd. for C_37_H_63_N_2_O_3_ [M + H]^+^ 583.48332, found 583.48116.

*[3-(piperazin-1-yl)propyl] 3-hydroxy-urs-12-en-28-oate (**9c**)*. 39% (purified by silica gel column chromatography with dichloromethane/methanol, 10:1), white solid. mp: 99.1–101.0 °C; ^1^H NMR (400 MHz, CDCl_3_): *δ* 5.22 (1H, s), 4.11–3.90 (2H, m), 3.21 (1H, dd, *J* = 10.8, 4.6 Hz), 3.15 (4H, s), 2.68 (4H, s), 2.53–2.38 (2H, m), 2.20 (1H, d, *J* = 11.2 Hz), 1.07 (3H, s), 0.98 (3H, s), 0.93 (3H, d, *J* = 5.6 Hz), 0.90 (3H, s), 0.84 (3H, d, *J* = 6.3 Hz), 0.77 (3H, s), 0.72 (3H, s); ^13^C NMR (100 MHz, CDCl_3_): *δ* 177.66, 138.37, 125.62, 79.14, 62.20, 55.31, 54.82, 52.97, 50.17, 48.21, 47.61, 43.76, 42.17, 39.65, 39.18, 38.99, 38.87, 38.71, 37.08, 36.88, 33.14, 30.76, 28.27, 28.08, 27.31, 25.95, 24.34, 23.66, 23.42, 21.31, 18.43, 17.24, 17.15, 15.79, 15.62. HR-MS *m/z* calcd. for C_37_H_63_N_2_O_3_ [M + H]^+^ 583.48332, found 583.48103.

*[4-(piperazin-1-yl)butyl] 3-hydroxy-urs-12-en-28-oate (**9d**)*. 38% (purified by silica gel column chromatography with dichloromethane/methanol, 10:1), white solid. mp: 100.0–102.5 °C; ^1^H NMR (400 MHz, CDCl_3_): *δ* 5.21 (1H, s), 4.06–3.91 (2H, m), 3.19–3.21 (5H, m), 2.70 (4H, s), 2.41 (2H, t, *J* = 7.0 Hz), 2.20 (1H, d, *J* = 11.2 Hz), 1.07 (3H, s), 0.98 (3H, s), 0.93 (3H, d, *J* = 5.8 Hz), 0.90 (3H, s), 0.84 (3H, d, *J* = 6.4 Hz), 0.77 (3H, s), 0.73 (3H, s); ^13^C NMR (100 MHz, CDCl_3_): *δ* 177.69, 138.31, 125.62, 79.10, 63.88, 57.60, 55.29, 52.99, 49.92, 48.17, 47.61, 43.76, 42.16, 39.63, 39.16, 38.98, 38.85, 38.71, 37.06, 36.87, 33.16, 30.76, 28.26, 28.08, 27.30, 26.42, 24.33, 23.65, 23.40, 23.27, 21.30, 18.41, 17.24, 17.15, 15.78, 15.60. HR-MS *m/z* calcd. for C_38_H_65_N_2_O_3_ [M + H]^+^597.49897, found 597.49693.

*[2-(4-methylpiperazin-1-yl)ethyl] 3-hydroxy-urs-12-en-28-oate (**9e**)*. 43% (purified by silica gel column chromatography with petroleum ether/ethyl acetate, 3:1), white solid. mp: 100.0–103.0 °C; ^1^H NMR (400 MHz, CDCl_3_): *δ* 5.22 (1H, s), 4.12 (2H, t, *J* = 6.0 Hz), 3.21 (1H, dd, *J* = 10.8, 4.7 Hz), 2.60 (10H, m), 2.33 (3H, d, *J* = 9.8 Hz), 2.20 (1H, d, *J* = 11.2 Hz), 1.07 (3H, s), 0.98 (3H, s), 0.93 (3H, d, *J* = 6.1 Hz), 0.90 (3H, s), 0.84 (3H, d, *J* = 6.4 Hz), 0.77 (3H, s), 0.74 (3H, s); ^13^C NMR (100 MHz, CDCl_3_): *δ* 177.48, 138.22, 125.72, 79.10, 61.97, 56.65, 55.33, 55.17, 53.29, 52.96, 48.10, 47.66, 46.05, 42.15, 39.67, 39.19, 39.00, 38.87, 38.74, 37.09, 36.79, 33.15, 30.79, 28.27, 28.10, 27.36, 24.34, 23.67, 23.41, 21.31, 18.43, 17.27, 17.16, 15.77, 15.59. HR-MS *m/z* calcd. for C_37_H_63_N_2_O_3_ [M + H]^+^583.48332, found 583.48100.

#### 3.2.5. General Procedure for Compounds **10** and **11**

Acetic anhydride (1.0 mL, 10.58 mmol) was added dropwise to the solution of UA (2.0 g, 4.38 mmol) in 15 mL pyridine under 0 °C. Subsequently, the mixture was stirred at room temperature for 15 h, poured into ice-cold water and filtered to give a white semisolid, 95% yield. The semisolid was dissolved with ethyl acetate (150 mL) and washed with 1 mol/L hydrochloric acid (60 mL), water (60 mL) and brine (60 mL), dried over Na_2_SO_4_ anhydrous, filtered, and then concentrated to afford 3-acetyl-UA. The mixture of 3-acetyl-UA (1.16 g, 2.33 mmol), K_2_CO_3_ (0.49 g, 3.55 mmol), and 2-chloroethanol (0.17 mL, 2.53 mmol) in 15 mL DMF was stirred at 60 °C for 3 h. Following the similar post-processing steps of **6, 10** was obtained as white powder, in 86% yield. Methanesulfonyl chloride (0.5 mL, 6.46 mmol) in pyridine (5 mL) was dropwise added to the solution of **10** (1.10 g, 2.03 mmol) in 15 mL pyridine under 10 °C. The mixture was stirred at room temperature for 3 h and treated with the similar post reaction treatment of 3-acetyl-UA to provide **11**, as off-white powder, in 94% yield.

#### 3.2.6. General Procedure for the Synthesis of Compounds **12a**–**c**

The mixture of **11** (1 equiv), K_2_CO_3_ (1 equiv), and piperazine (6 equiv), homopiperazine (6 equiv) or morpholine (2 equiv) in DMF was stirred at 60 °C for 30 min, poured into ice-cold water, stirred, and filtered. The off-white solid was dissolved in ethyl acetate and washed with water and brine, dried over Na_2_SO_4_ anhydrous, filtered, evaporated, and purified by silica gel column chromatography to yield **12a**–**c**.

*[2-(piperazin-1-yl)ethyl] 3-acetoxy-urs-12-en-28-oate (**12a**)*. 73% (purified by silica gel column chromatography with dichloromethane/methanol, 10:1), white solid. mp: 80.1–82.9 °C; ^1^H NMR (400 MHz, CDCl_3_): *δ* 5.21 (1H, s), 4.52–4.44 (1H, m), 4.16–4.04 (2H, m), 3.10 (4H, s), 2.72 (4H, s), 2.63 (2H, t, *J* = 5.6 Hz), 2.18 (1H, d, *J* = 12.5 Hz), 2.04 (3H, s), 1.06 (3H, s), 0.93 (6H, d, *J* = 5.8 Hz), 0.85 (9H, d, *J* = 4.9 Hz), 0.72 (3H, s); ^13^C NMR (100 MHz, CDCl_3_) *δ* 177.51, 171.19, 138.26, 125.62, 81.03, 61.59, 58.52, 56.94, 55.39, 52.97, 52.78, 48.13, 47.56, 45.13, 42.14, 39.67, 39.16, 38.99, 38.39, 37.79, 36.97, 36.83, 33.07, 30.75, 28.19, 28.07, 24.32, 23.66, 23.62, 23.41, 21.46, 21.29, 18.56, 18.31, 17.25, 17.18, 16.86, 15.65. HR-MS *m/z* calcd. for C_38_H_63_N_2_O_4_ [M + H]^+^ 611.47823, found 611.47836.

*[2-(Homopiperazine-1-yl)ethyl] 3-acetoxy-urs-12-en-28-oate (**12b**)*. 71% (purified by silica gel column chromatography with dichloromethane/methanol, 10:1), white solid. mp: 76.5–78.0 °C. ^1^H NMR (400 MHz, CDCl_3_): *δ* 5.22 (1H, s), 4.53–4.44 (1H, m), 4.16 (2H, s), 3.38 (4H, s), 3.20 (2H, s), 2.94–3.00 (4H, m), 2.04 (3H, s), 1.06 (3H, s, CH), 0.94 (6H, d, *J* = 3.8 Hz), 0.85 (9H, d, *J* = 4.6 Hz), 0.72 (3H, s); ^13^C NMR (100 MHz, CDCl_3_): *δ* 177.52, 171.17, 138.22, 125.64, 81.01, 61.84, 56.44, 55.38, 53.97, 53.00, 51.16, 48.16, 47.55, 46.85, 44.47, 42.15, 39.65, 39.14, 38.97, 38.38, 37.79, 36.97, 36.87, 33.08, 30.72, 28.19, 28.08, 25.31, 24.35, 23.65, 23.60, 23.41, 21.45, 21.28, 18.31, 17.27, 17.18, 16.86, 15.67. HR-MS *m/z* calcd. for C_39_H_65_N_2_O_4_ [M + H]^+^ 625.49388, found 625.49126.

*(2-morpholinoethyl) 3-acetoxy-urs-12-en-28-oate (**12c**)*. 75% (purified by silica gel column chromatography with petroleum ether/ ethyl acetate, 3:1), white solid. mp: 63.1–65.1 °C. ^1^H NMR (400 MHz, CDCl_3_): *δ* 5.22 (1H, s), 4.54–4.44 (1H, m), 4.27 (2H, s), 3.82 (4H, s), 2.19 (1H, d, *J* = 11.2 Hz), 2.04 (3H, s), 1.07 (3H, s), 0.94 (6H, d, *J* = 5.5 Hz), 0.85 (9H, d, *J* = 4.9 Hz), 0.74 (3H, s); ^13^C NMR (100 MHz, CDCl_3_): *δ* 177.37, 171.16, 138.24, 125.72, 81.00, 66.21, 56.95, 55.39, 53.52, 52.97, 48.17, 47.54, 42.15, 39.69, 39.16, 38.99, 38.38, 37.79, 36.98, 36.82, 33.06, 30.72, 29.82, 28.19, 28.04, 24.34, 23.65, 23.63, 23.39, 21.45, 21.28, 18.30, 17.32, 17.18, 16.86, 15.64. HR-MS *m/z* calcd. for C_38_H_62_NO_5_ [M + H]^+^ 612.46225, found 612.45986.

#### 3.2.7. Preparation of Compound **13**

The mixture of **9a** (500 mg, 0.88 mmol) and PCC (190 mg, 0.88 mmol) in 80 mL chloroform was stirred at 0 °C for 10 h, then isopropanol (1.0 mL, 13.1 mmol) was added and stirred at room temperature for 1 h. The mixture was washed with water (40 mL) and brine (2 × 40 mL), dried over Na_2_SO_4_ anhydrous, filtered, concentrated, and purified by silica gel column chromatography with dichloromethane/methanol (10:1) to obtain *[2-(Piperazin-1-yl)ethyl] 3-oxo-urs-12-en-28-oate (**13**)*. 69%, off-white powder. mp:144.4–146.8 °C; ^1^H NMR (400 MHz, CDCl_3_): *δ* 5.24 (1H, s), 4.18–4.04 (2H, m), 3.19 (4H, s), 2.81 (4H, s), 2.65 (2H, t, *J* = 5.4 Hz), 2.59-2.47 (1H, m), 2.34–2.39 (1H, m), 2.23–2.14 (1H, m), 1.08 (6H, s), 1.03 (6H, s), 0.94 (3H, d, *J* = 5.5 Hz), 0.85 (3H, d, *J* = 6.3 Hz), 0.77 (3H, s); ^13^C NMR (100 MHz, CDCl_3_): *δ* 218.00, 177.42, 138.33, 125.48, 61.20, 56.39, 55.33, 53.06, 49.91, 48.20, 47.52, 46.82, 43.72, 42.27, 39.60, 39.38, 39.15, 38.95, 36.83, 36.77, 34.29, 32.65, 30.70, 28.06, 26.67, 24.30, 23.56, 21.62, 21.24, 19.69, 17.23, 17.13, 15.36. HR-MS *m/z* calcd. for C_36_H_59_N_2_O_3_ [M + H]^+^567.45202, found 567.44977.

#### 3.2.8. General Procedure for Synthesizing Compounds **14a** and **14b**

The mixture of **12a** (300 mg, 0.49 mmol), K_2_CO_3_ (70 mg, 0.51 mmol), and 4-fluorobenzyl bromide (120 mg, 0.63 mmol) or 2-nitrobenzyl bromide (130 mg, 0.60 mmol) in DMF (10 mL) was stirred at 60 °C for 1 h, the mixture was poured into 50 mL ice-cold water and then filtered. The white solid was dissolved in ethyl acetate (100 mL) and washed with water (50 mL) and brine (2 × 50 mL), dried over Na_2_SO_4_ anhydrous, filtered, evaporated, and purified by silica gel column chromatography with dichloromethane/methanol (20:1) to obtain **14a** and **14b**.

*{2-[4-(4-fluorobenzyl)piperazin-1-yl]ethyl} 3-acetoxy-urs-12-en-28-oate (**14a**)*. 45% as white solid. mp: 140.0–141.8 °C; ^1^H NMR (400 MHz, CDCl_3_): *δ* 7.76–7.58 (2H, m), 7.12 (2H t, *J* = 8.2 Hz), 5.16 (1H, s), 4.51–4.43 (1H, m), 4.08 (2H, s), 2.73–3.90 (10H, m), 2.09 (1H, d, *J* = 11.3 Hz), 2.04 (3H, s), 1.04 (3H, d, *J* = 7.4 Hz), 0.92 (6H, d, *J* = 10.4 Hz), 0.88–0.81 (9H, m), 0.65 (3H, d, *J* = 13.5 Hz); ^13^C NMR (100 MHz, CDCl_3_): *δ* 176.88, 171.17, 164.24 (d, *J* = 252.6 Hz), 138.14, 136.01 (d, *J* = 8.5 Hz), 130.81 (d, *J* = 7.6 Hz), 125.92, 122.57, 116.74 (d, *J* = 21.6 Hz), 115.67 (d, *J* = 21.4 Hz), 80.89, 61.04, 57.23, 56.48, 55.33, 53.12, 48.38, 47.42, 46.25, 46.17, 42.18, 39.65, 39.08, 39.04, 38.35, 37.77, 36.93, 36.78, 32.96, 30.50, 28.17, 27.97, 24.36, 23.56 (d, *J* = 4.5 Hz), 23.38, 21.43, 21.16, 18.22, 17.47, 17.17, 16.84, 15.62. HR-MS *m/z* calcd. for C_45_H_68_FN_2_O_4_ [M + H]^+^ 719.51576, found 719.51562.

*{2-[4-(2-nitrobenzyl)piperazin-1-yl]ethyl} 3-acetoxy-urs-12-en-28-oate (**14b**)*. 43%, white solid. mp: 83.0–85.0 °C; ^1^H NMR (400 MHz, CDCl_3_): *δ* 7.80 (1H, d, *J* = 7.9 Hz), 7.60–7.47 (2H, m), 7.40 (1H, t, *J* = 7.5 Hz), 5.21 (1H, s), 4.55–4.41 (1H, m), 4.17 (2H, s), 3.80 (2H, s), 2.61 (8H, m), 2.19 (1H, d, *J* = 11.3 Hz), 2.04 (3H, s), 1.06 (3H, s), 0.94 (6H, d, *J* = 4.9 Hz), 0.86 (9H, d, *J* = 4.0 Hz), 0.74 (3H, d, *J* = 10.5 Hz); ^13^C NMR (100 MHz, CDCl_3_): *δ* 177.44, 171.19, 149.98, 138.23, 132.49, 131.16, 128.21, 125.67, 124.57, 118.04, 81.04, 59.05, 56.49, 55.40, 53.35, 52.94, 48.12, 47.57, 42.14, 39.68, 39.17, 38.97, 38.39, 37.80, 36.98, 36.79, 33.07, 30.75, 28.21, 28.06, 24.32, 23.67, 23.63, 23.41, 21.48, 21.31, 18.31, 17.29, 17.19, 16.88, 15.66. HR-MS *m/z* calcd. for C_45_H_68_N_3_O_6_ [M + H]^+^ 746.51062, found 746.51035.

#### 3.2.9. General Procedure for Synthesizing Compounds **17** and **18**

Compound **17** was synthesized from compound **1** according to the literature method [25]. **18** was prepared from **17** according to the synthetic procedure of **12a** and then purified by silica gel column chromatography with dichloromethane/methanol (15:1) to afford **18**.

*Urs-12-en-[2,3-c]*[1,2,5]*oxadiazole oxide-28-oic acid (**17**)*. 60%, white solid. mp: >286 °C; ^1^H NMR (400 MHz, CDCl_3_): *δ* 5.29 (1H, s), 2.72 (1H, d, *J* = 16.6 Hz), 2.23 (1H, d, *J* = 11.6 Hz), 2.17 (3H, s), 1.41 (3H, s), 1.30 (3H, s), 1.10 (3H, s), 0.95 (3H, d, *J* = 5.9 Hz), 0.91 (3H, s), 0.86 (3H d, *J* = 6.3 Hz), 0.82 (1H, s); ^13^C NMR (100 MHz, CDCl_3_): *δ* 184.01, 163.66, 138.25, 125.04, 112.03, 52.71, 52.65, 48.14, 46.01, 42.27, 39.63, 39.20, 38.90, 38.40, 36.75, 35.17, 34.72, 32.04, 30.71, 28.08, 24.10, 23.92, 23.55, 23.29, 21.27, 18.91, 17.09, 16.72, 16.30.HR-MS *m/z* calcd. for C_30_H_45_O_4_N_2_ [M + H]^+^ 497.33738, found 497.33572.

*[2-(Piperazin-1-yl)ethyl)] urs-12-en-[2,3-c]*[1,2,5]*oxadiazole oxide-28-oate (**18**)*. 47%, white powder. mp: 134.1–136.8 °C; ^1^H NMR (400 MHz, CDCl_3_): *δ* 5.29 (1H, s, H-12), 4.12 (2H, t, *J* = 5.7 Hz, CH_2_), 3.02–3.05 (4H, m), 2.64 (4H, d, *J* = 4.1 Hz), 2.62 (2H, d, *J* = 5.8 Hz), 2.24 (1H, d, *J* = 11.1 Hz), 1.41 (3H, s), 1.33 (3H, s), 1.09 (3H, s), 0.94 (3H, d, *J* = 5.7 Hz), 0.91 (3H, s), 0.86 (3H, d, *J* = 6.3 Hz), 0.79 (3H, s); ^13^C NMR (100 MHz, CDCl_3_): *δ* 177.38, 163.72, 138.49, 124.84, 112.02, 61.77, 57.17, 54.01, 53.02, 52.77, 48.16, 46.01, 45.79, 42.36, 39.69, 39.26, 38.96, 38.39, 36.72, 35.20, 34.73, 32.16, 30.75, 28.07, 24.27, 23.96, 23.48, 23.33, 21.26, 18.97, 17.13, 16.92, 16.32. HR-MS *m/z* calcd. for C_36_H_57_O_4_N_4_ [M + H]^+^ 609.43743, found 609.43526.

### 3.3. Biology

#### 3.3.1. Cell Culture and Treatment

MKN45, Hela cell (the Shanghai Institute of Life Sciences) were cultivated in DMEM media (Hyclone, Thermo Fisher Scientific, Waltham, MA, USA) supplemented with 10% fetal bovine serum (Life Technology, Thermo Fisher Scientific, Waltham, MA, USA), 100 U/mL penicillin, and l00 μg/mL streptomycin, and grown in a humidified incubator (37 °C, 5% CO_2_). All of the cells were harvested in the exponential growth phase for assays.

#### 3.3.2. MTT Assay

All of the tested compounds were dissolved in DMSO and subsequently diluted in the culture medium before treatment of the cultured cells. When the cells were 80–90% confluent, they were harvested by treatment with a solution containing 0.25% trypsin, thoroughly washed, and resuspended in supplemented growth medium. Cells (2 × 10^3^/well) were plated in 100 μL of medium/well in 96-well plate. After incubations overnight, the cells were treated with different concentrations of the compounds in DMEM with 10% FBS for 72 h. In parallel, the cells that were treated with DMSO served as negative control and CDDP (cisplatin) as positive control. Finally, 20 μL of MTT was added and the cells were incubated for 4 h. The MTT-formazan that formed by metabolically viable cells was dissolved in 100 μL of DMSO for 10 min. The absorbance was then measured at 570 nm with a microplate reader, in which the cell survival rates were calculated and the IC_50_ was calculated following our previous method [26,27].

#### 3.3.3. Flow Cytometric Analysis

To determine the effect of compound **14a** on the cell cycle, the cells were seeded in six-well plates at a density of 2 × 10^5^ cells/mL for 24 h. After incubation, the cells were treated with indicated concentrations of compound **14a** for 24 h. Subsequently, the cells were collected, washed, and fixed in 70% ethanol in PBS at −20 °C. After overnight, fixed cells were pelleted and stained with cell cycle analysis reagent propidium iodide solution as per the manufacturer instructions for 30 min at 37 °C in dark, and about 5000 events were analysed on a FACScan cytometry(C6 Plus, Becton Dickinson, San Jose, CA, USA) following published protocol [28].

#### 3.3.4. Western Blotting Assay

The Hela cell were treated with **14a** at three concentrations: 0, 2, 5 µM. After treatment, the cells were washed with PBS and kept in −80 °C until further handing. Western blotting was then performed, as previously described. Briefly, total cell extracts were collected by RIPA buffer and electrophoresed on 8% or 10% SDS-polyacrylamide gels and then transferred to PVDF membranes (Millipore, Temecula, CA, USA). The membranes were blocked and then incubated overnight at 4 °C with primary antibodies to Bax (2772, Cell Signaling Technology, Boston, MA, USA), Bcl2 (2876, Cell Signaling Technology, Boston, MA, USA), PARP (46D11, Cell Signaling Technology, Boston, MA, USA), and β-actin (C4, Santa Cruz Biotechnology, Dallas, TX, USA). The member was developed and visualized by Image Quant LAS4000 (GE Healthcare, Boston, MA, USA) [29].

#### 3.3.5. Xenograft Studies

Nude mice (6−8 weeks old, BALB/c, female) were used to establish the xenograft tumors following the published protocol [30]. Briefly, Hela cells (5 × 10^6^) were implanted in the dorsal region of recipient mice by means of subcutaneous injection. Once a tumor had reached around 300 mm^3^ in size, the mice were randomized into three groups as control, **14a** (50 mg/kg) and cisplatin (3 mg/kg) with five mice per group. The animals were administrated by intraperitoneal injection for cisplatin and administrated by gavage for **14a**. Tumor growth and body weight were measured every three days during the treatment. At the end of the treatment, the mice were sacrificed and tumors were removed and weighed. The use of animals was approved by the Animal Experimentation Ethics Committee of Yantai University in accordance with the guidelines for ethical conduct in the care and use of animals (Ethical code YT-YX-1817).

## 4. Conclusions

In summary, nineteen nitrogen-bearing heterocyclic UA analogues have been synthesized in this work. The antiproliferative activity against Hela and MKN45 cell lines in vitro were evaluated. The results showed that compounds bearing thiazole, triazole or tetrazole groups (**5a**–**e** and **7**) displayed moderate antitumor activity. **9a**, **9c**, **12a**, **12b**, **13**, **14a**, **18** (containing a piperazine or homopiperazine moiety) exhibited promising results against the cancer cell lines that were tested here, with IC_50_ values from 2.1–9.8 µM. In addition, an analysis of the structure-activity relationships revealed that a number of factors remarkably affected the cytotoxicity. These included acetylation of 3-OH, the type of nitrogen heterocycle, the linkers’ length between 28-COOH and nitrogen heterocycles, as well as different substituents in the piperazine. **14a**, which is one of the most active compounds, decreased the apoptosis regulator (BCL2/BAX) ratio, which subsequently disrupted the mitochondrial potential and induced apoptosis and significantly suppressed the growth of Hela xenografts in nude mice.

The anti-cancer activity of the newly synthesized ursolic acid analogs is encouraging and may provide a starting point for further structural optimization. Further pharmacokinetic study for these compounds is planned.

## Figures and Tables

**Table 1:** Cytotoxic activities of the compounds against Hela and MKN45 cell lines.

Compound	Inhibition Rates ^a^		IC_50_ (µM) ^b^	
	Hela	MKN45	Hela	MKN45
UA	13%	18%	15.1 ± 2.7	16.7 ± 1.4
**5a**	1%	31%	>20	>20
**5b**	2%	35%	>20	>20
**5c**	16%	45%	>20	>20
**5d**	3%	31%	NT^c^	NT
**5e**	2%	32%	NT	NT
**7**	1%	35%	NT	NT
**9a**	59%	51%	9.8 ± 1.8	7.4 ± 1.9
**9b**	34%	29%	>20	>20
**9c**	61%	55%	9.3 ± 0.5	7.9 ± 0.2
**9d**	9%	10%	16.2 ± 0.8	19.3 ± 0.3
**9e**	24%	27%	NT	NT
**12a**	81%	77%	6.4 ± 0.3	6.4 ± 0.1
**12b**	89%	75%	7.3 ± 0.2	6.2 ± 0.5
**12c**	8%	0%	NT	NT
**13**	58%	61%	8.5 ± 0.3	8.9 ± 0.1
**14a**	89%	84%	2.6 ± 1.1	2.1 ± 0.3
**14b**	16%	7%	NT	NT
**17**	3%	3%	NT	NT
**18**	90%	86%	3.9 ± 0.6	4.5 ± 0.2
CDDP ^d^	77%	62%	15.1 ± 0.9	2.8 ± 0.1

^a^ Inhibition rate of cells treated with 10 μM concentration for 72 h. ^b^ The concentration (μM) of compound that inhibited 50% of cell growth at 72 h after treatment. ^c^ Not tested. ^d^ Cisplatin, positive control.

**Table 2:** The anti-tumor activity of **14a** against the growth of Hela in nude mice^a^.

Group (mg/kg)	Number (Initial/End)	Body Weight (g)		Tumor Weight (g)	
		Initial	End	g	IR (%)
**Control**	5/5	20.52 ± 0.99	20.56 ± 2.11	2.12 ± 0.40	
**Cisplatin** 3	5/5	20.80 ± 1.34	21.70 ± 1.32	0.88 ± 0.12 *	58.49
**14a 50**	5/5	21.08 ± 1.04	20.48 ± 0.89	1.20 ± 0.59 *	43.40

*^a^* Data are expressed as the mean ±SD (n = 5). *p < 0.05, compared with control.

## Supplementary Materials

The following are available online at https://www.mdpi.com/1420-3049/24/5/877/s1, ^1^H-NMR, ^13^C-NMR and HR-MS spectra of compounds.
