# Supplementary material for: Design, Synthesis, and Biological Evaluation of Novel Nitrogen Heterocycle-Containing Ursolic Acid Analogs as Antitumor Agents
Source: Molecules. 2019 Mar 1;24(5):877. doi: 10.3390/molecules24050877 (PMC6429512; doi:10.3390/molecules24050877)
Supplement: Supplementary file 1 [file molecules-24-00877-s001.pdf]

Supplementary data

^{1}H NMR, ^{13}CNMR and HRMS spectra of compounds

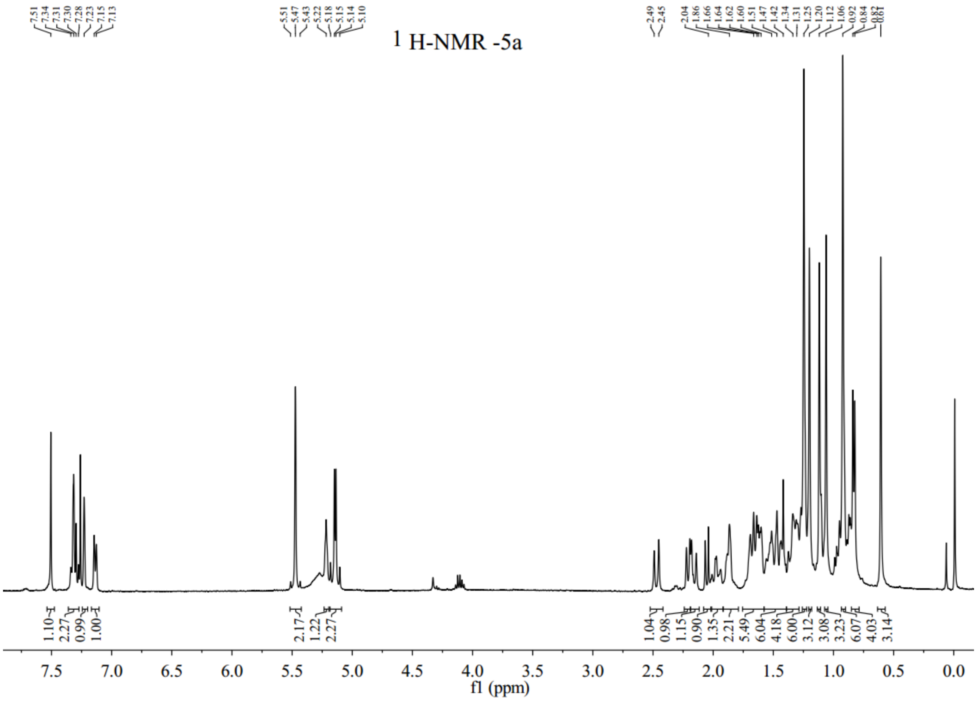

Figure 1. ^{1}H NMR spectra of **5a.**

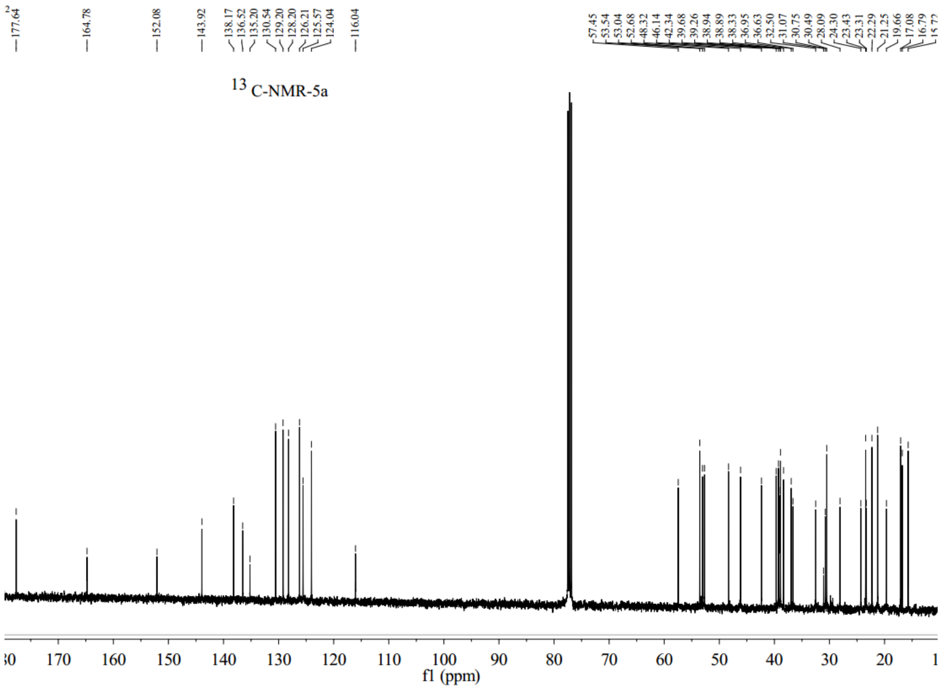

Figure 2. ^{13}C NMR spectra of **5a.**

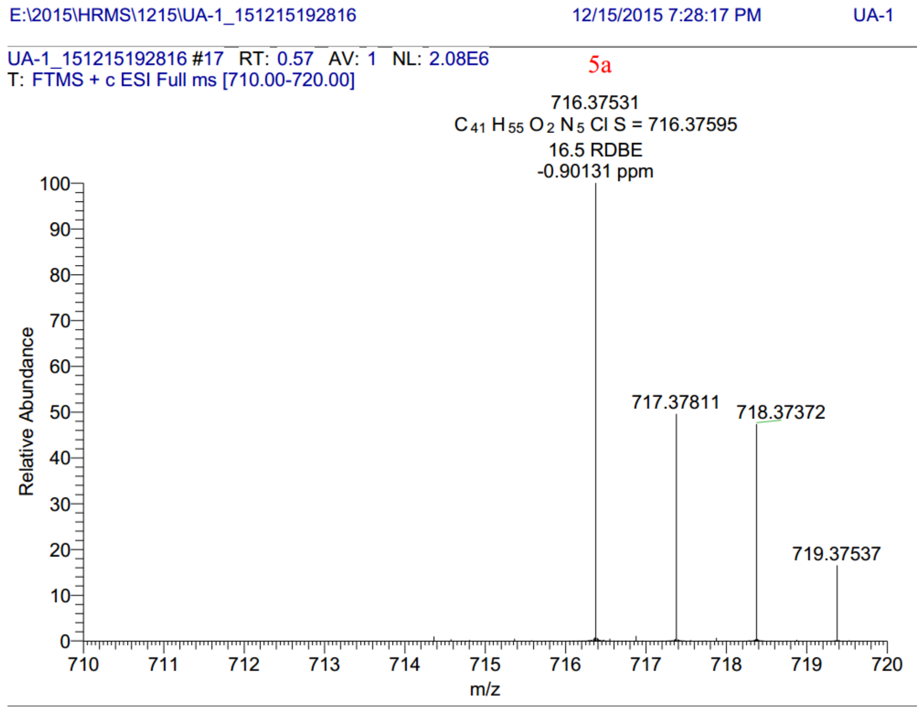

Figure 3. HR-MS spectra of **5a.**

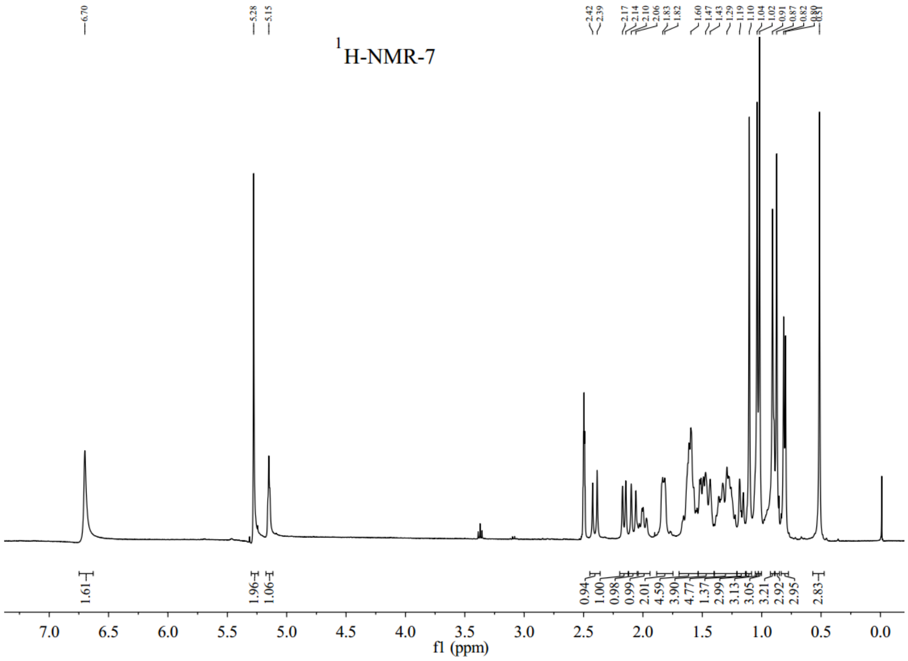

Figure S4. ^{1}H NMR spectra of **7**

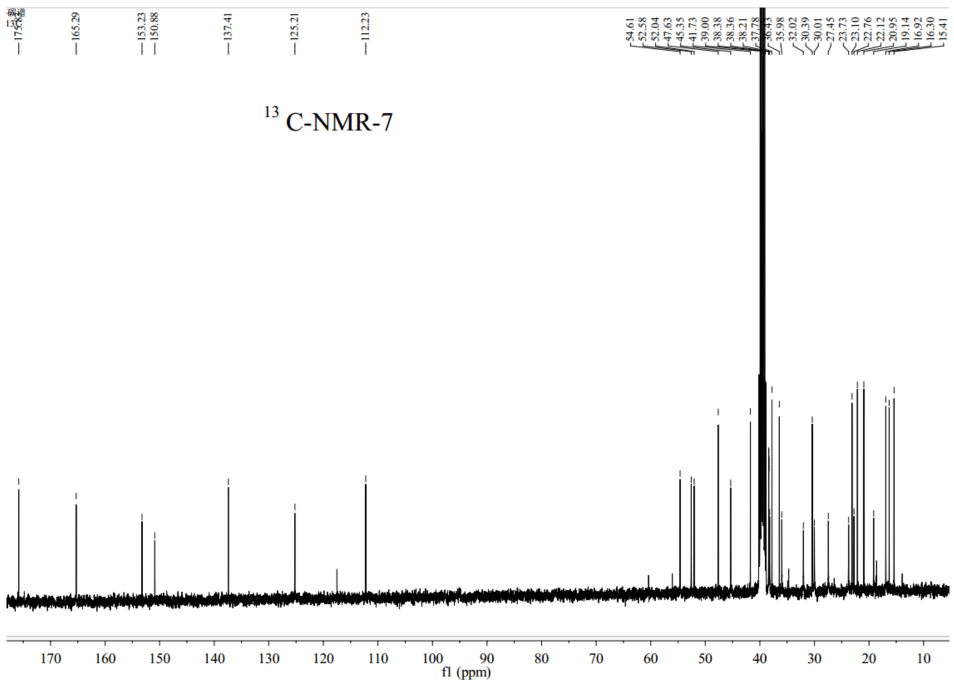

Figure 5. ^{13}C NMR spectra of **7.**

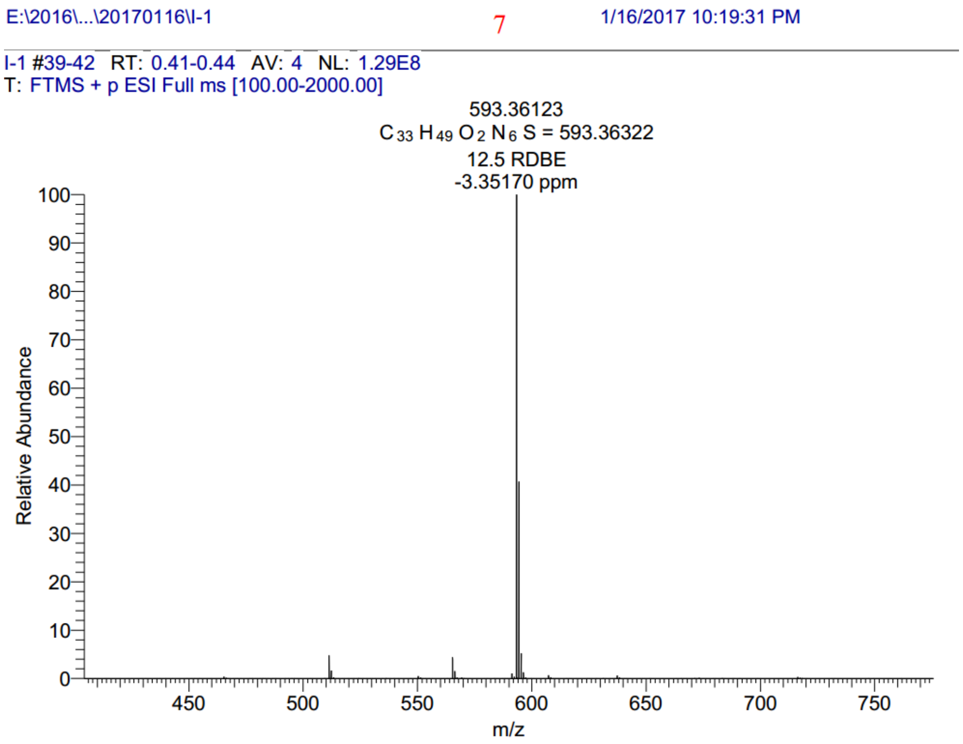

Figure S6. HRMS spectra of **7**

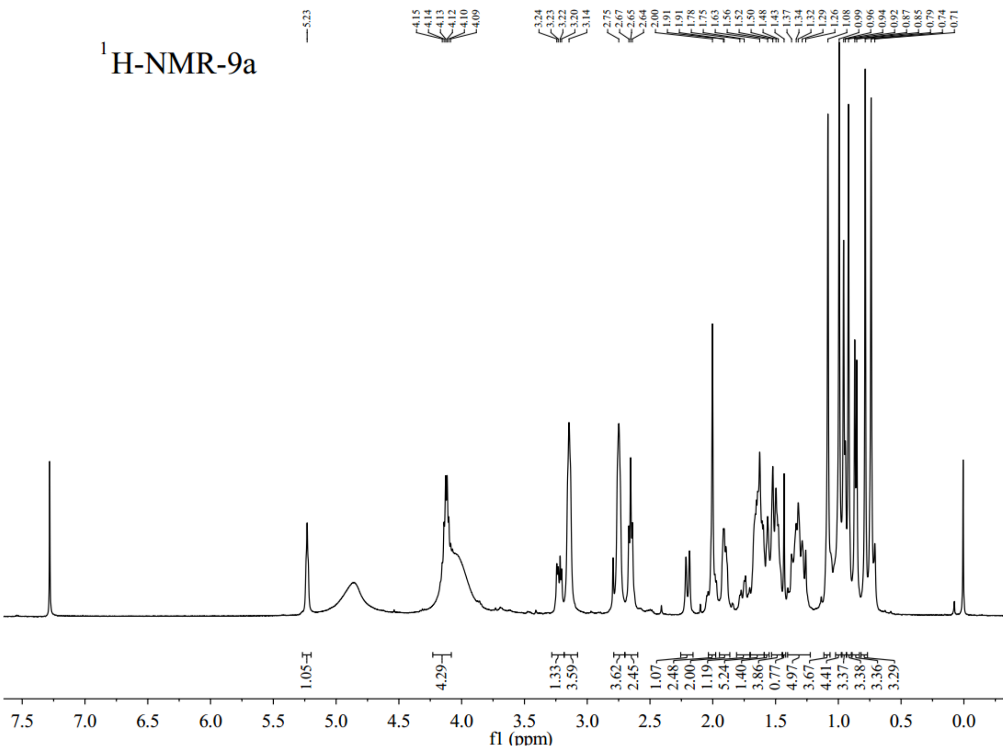

Figure 7. ^{1}H NMR spectra of **9a.**

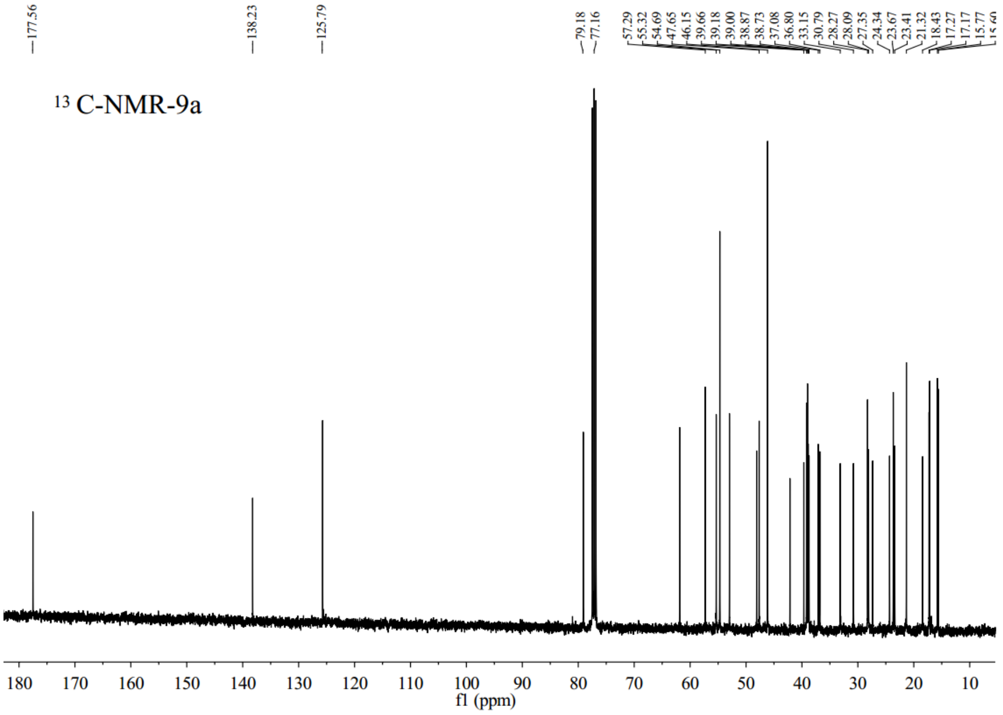

Figure 8. ^{13}C NMR spectra of **9a.**

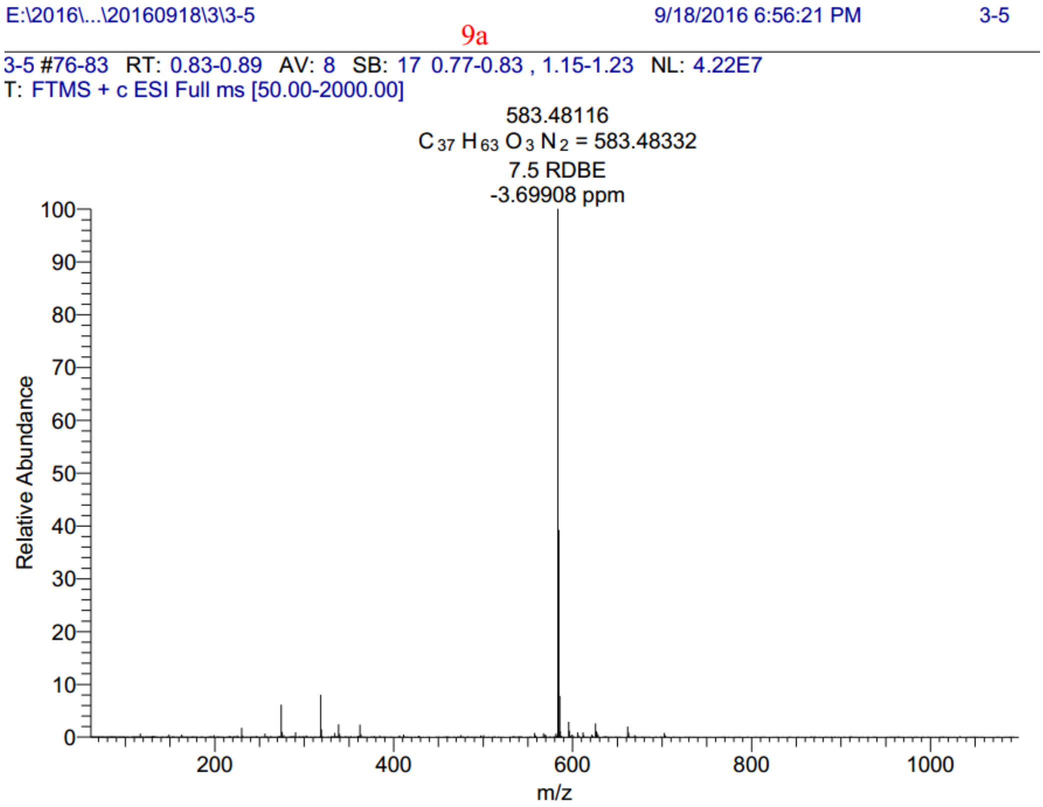

Figure 9. HRMS spectra of **9a.**

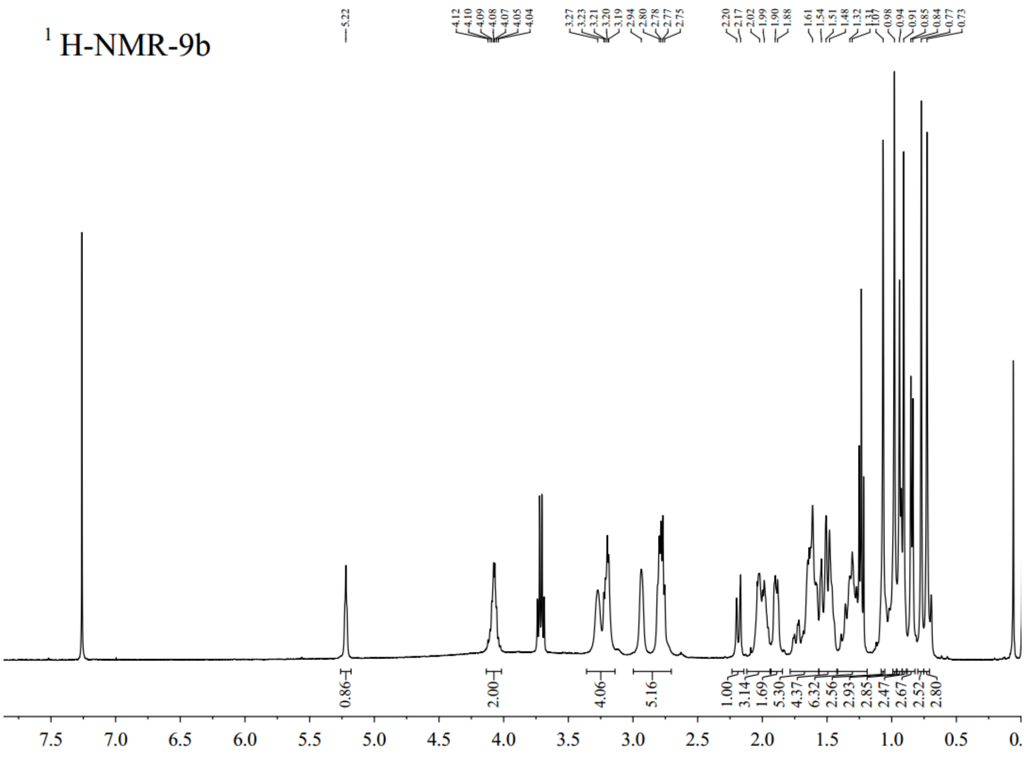

Figure S10. ^{1}H NMR spectra of **9b**

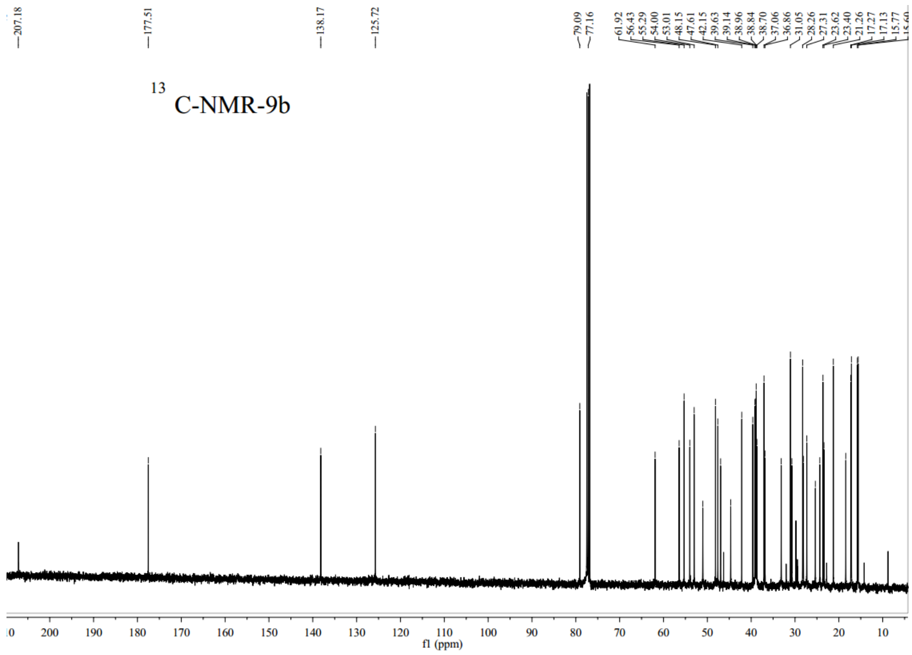

Figure S11. ^{13}C NMR spectra of **9b**

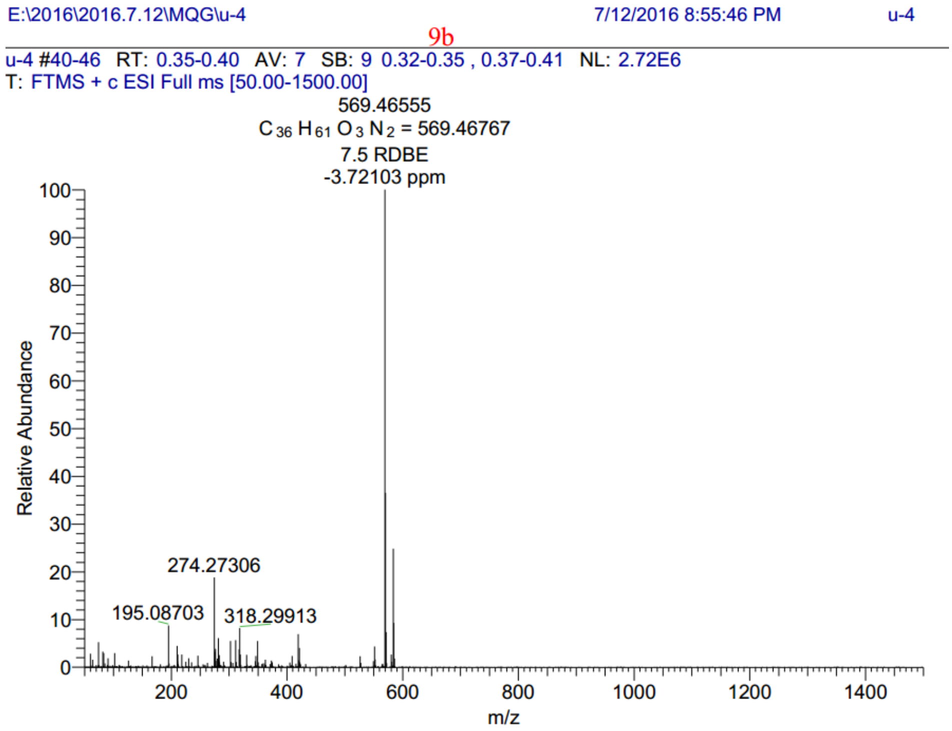

Figure S12. HRMS spectra of **9b**

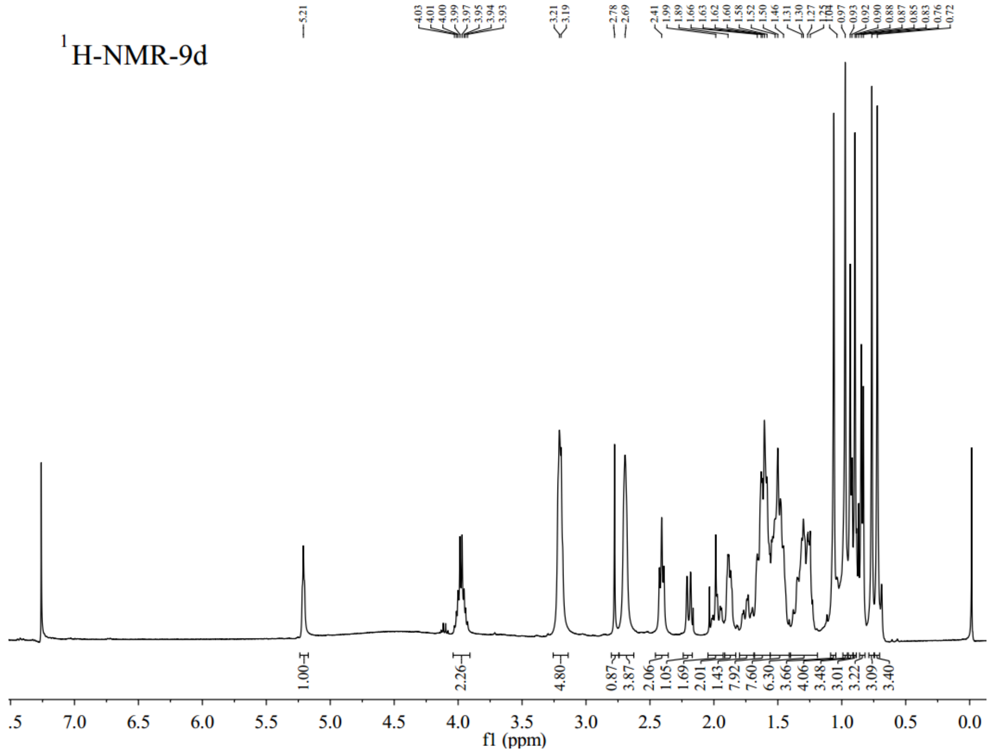

Figure S13. ^{1}H NMR spectra of **9d**

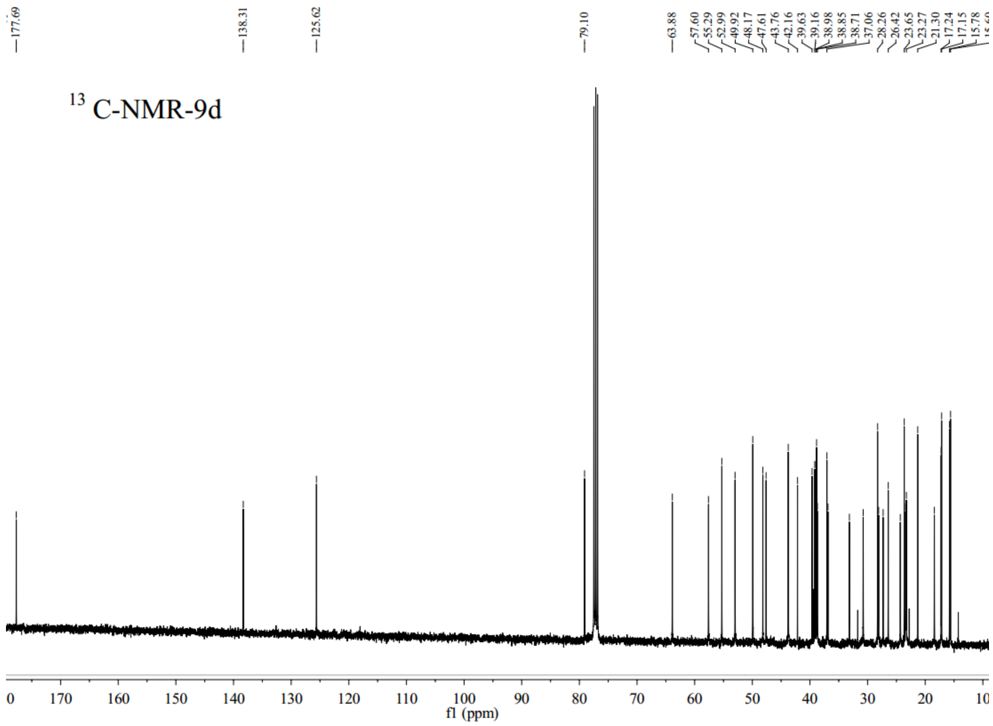

Figure S14. ^{13}C NMR spectra of **9d**

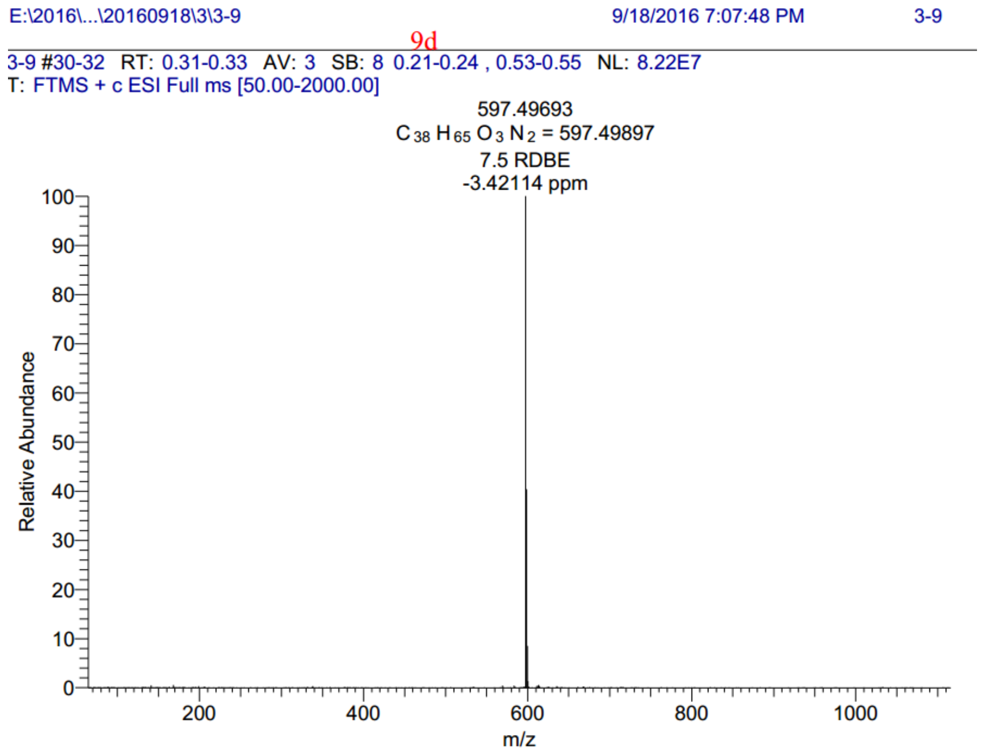

Figure S15. HRMS spectra of **9d**

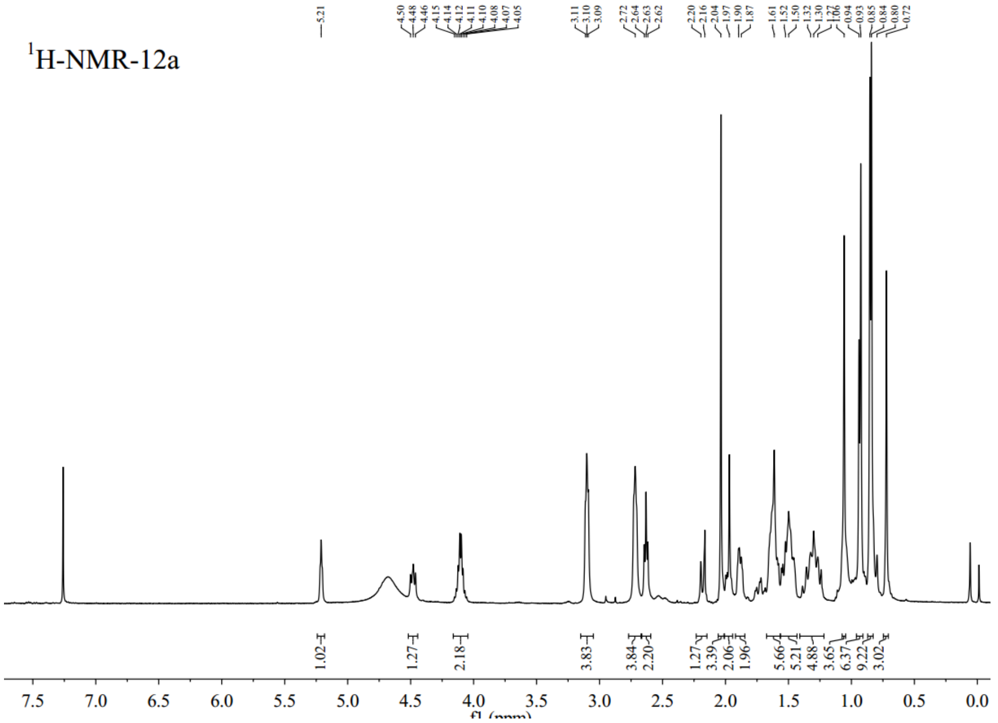

Figure S16. ^{1}H NMR spectra of **12a**

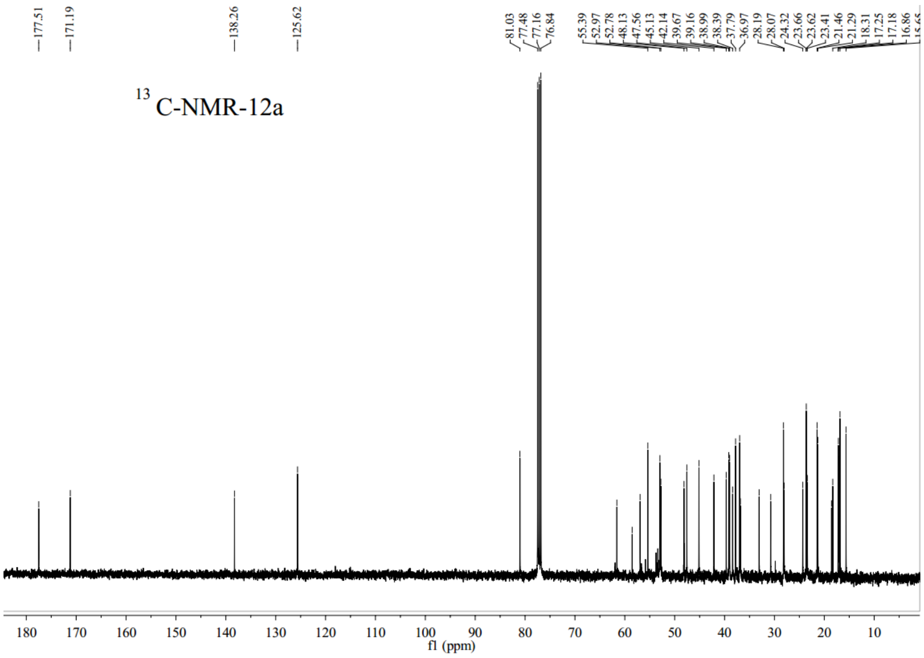

Figure S17. ^{13}C NMR spectra of **12a**

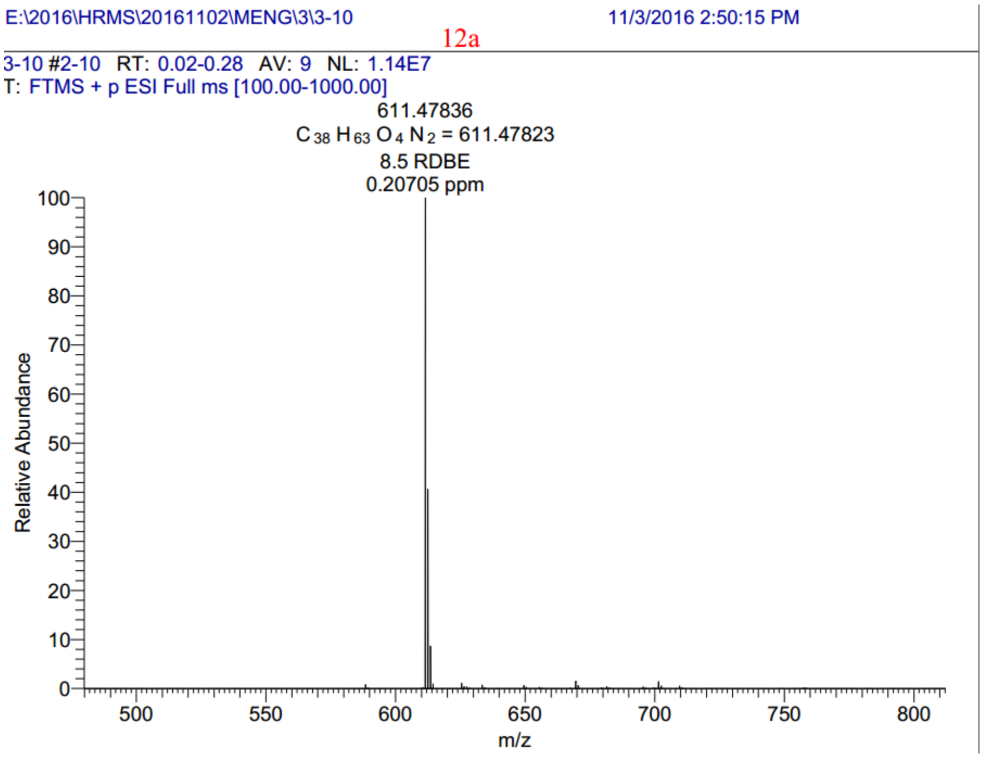

Figure S18. HRMS spectra of **12a**

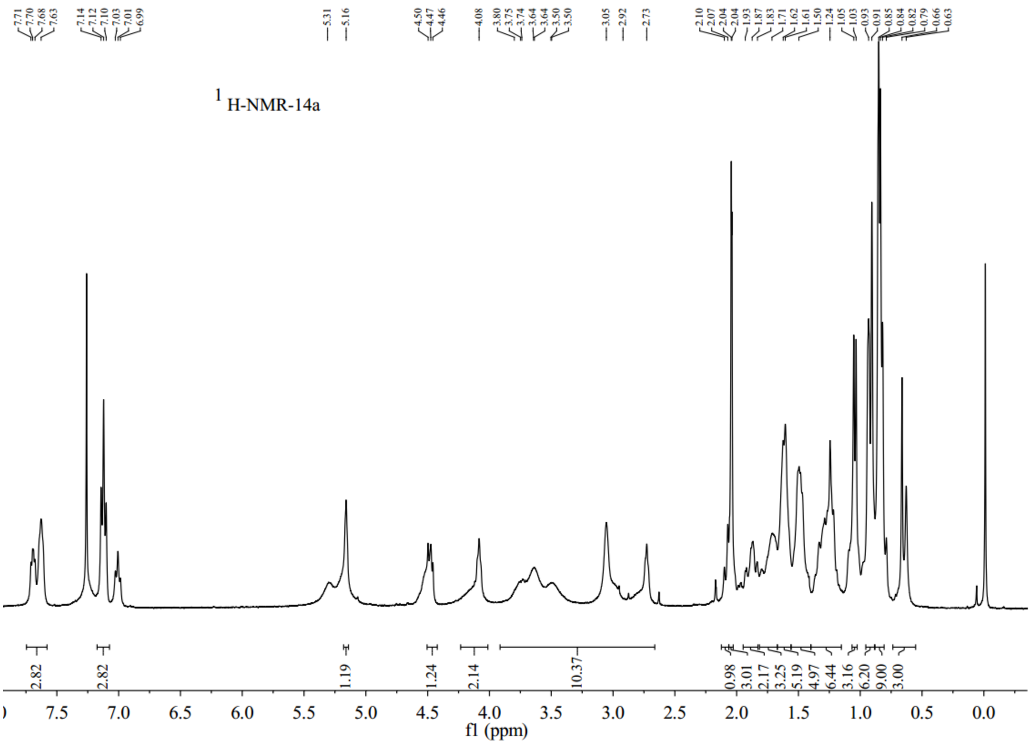

Figure S19. ^{1}H NMR spectra of **14a**

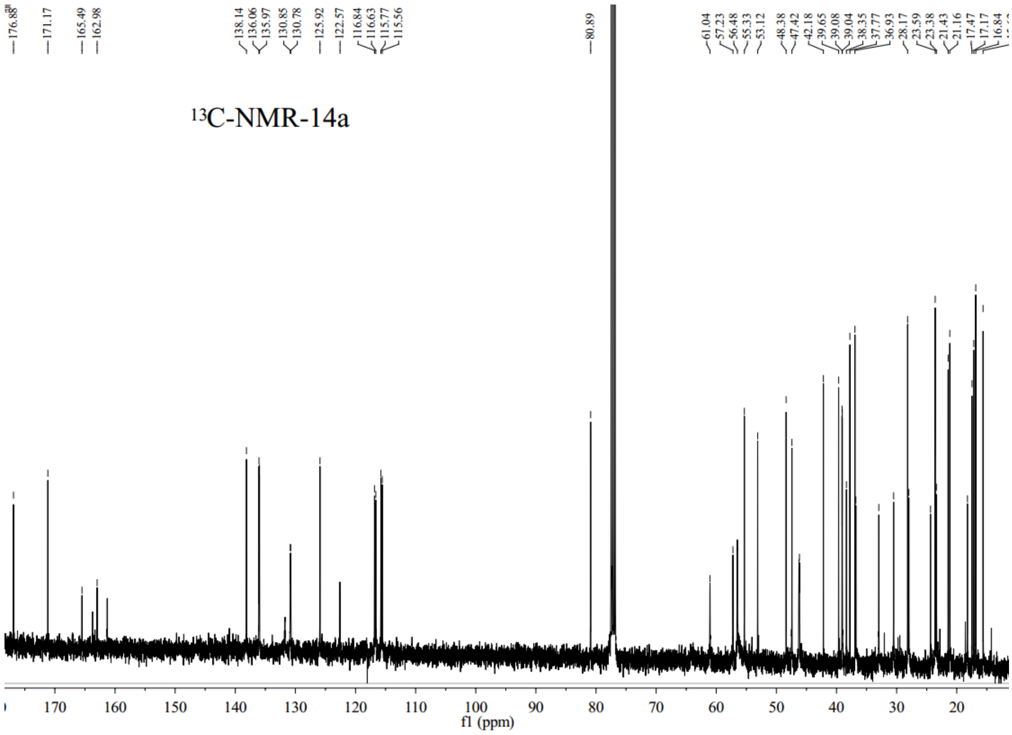

Figure S20. ^{13}C NMR spectra of **14a**

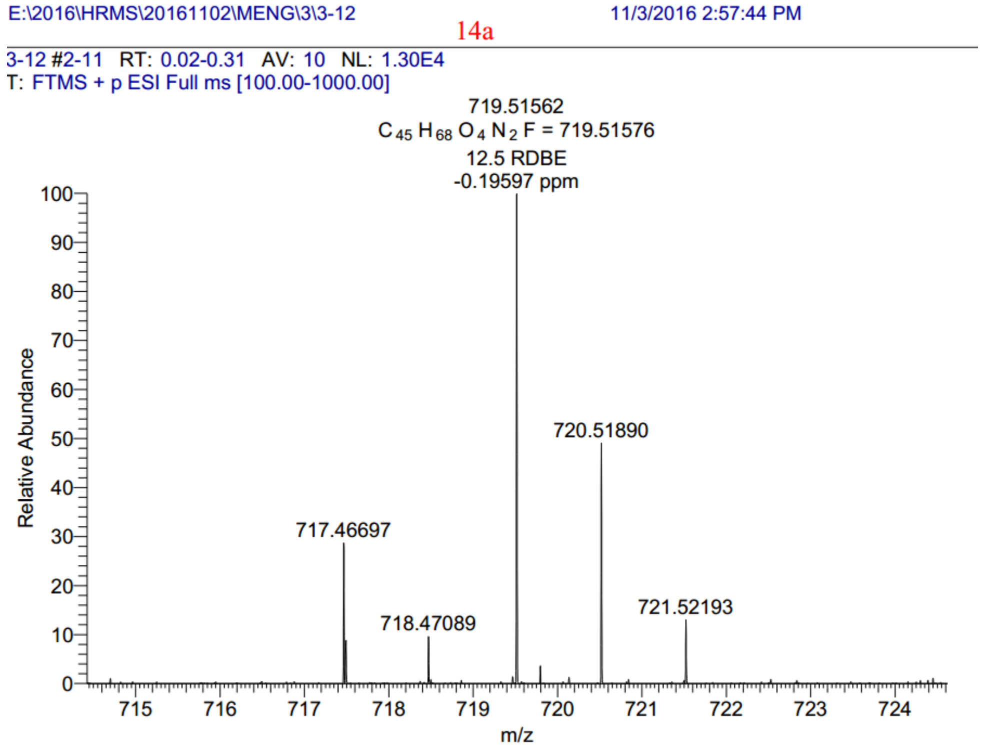

Figure S21. HRMS spectra of **14a**

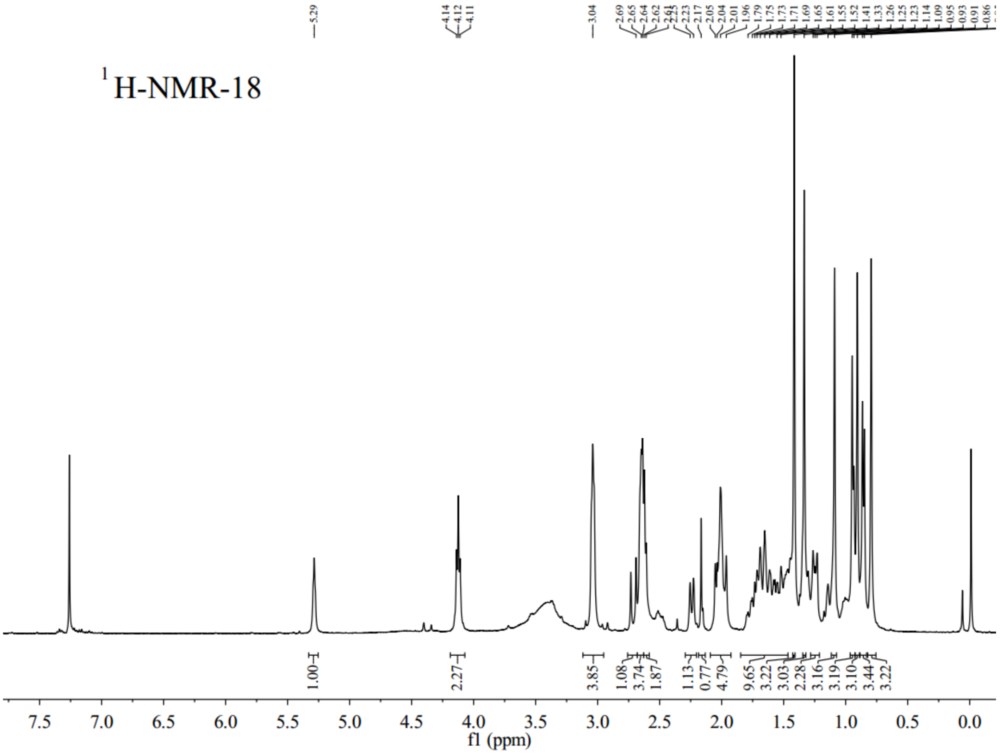

Figure S22. ^{1}H NMR spectra of **18**

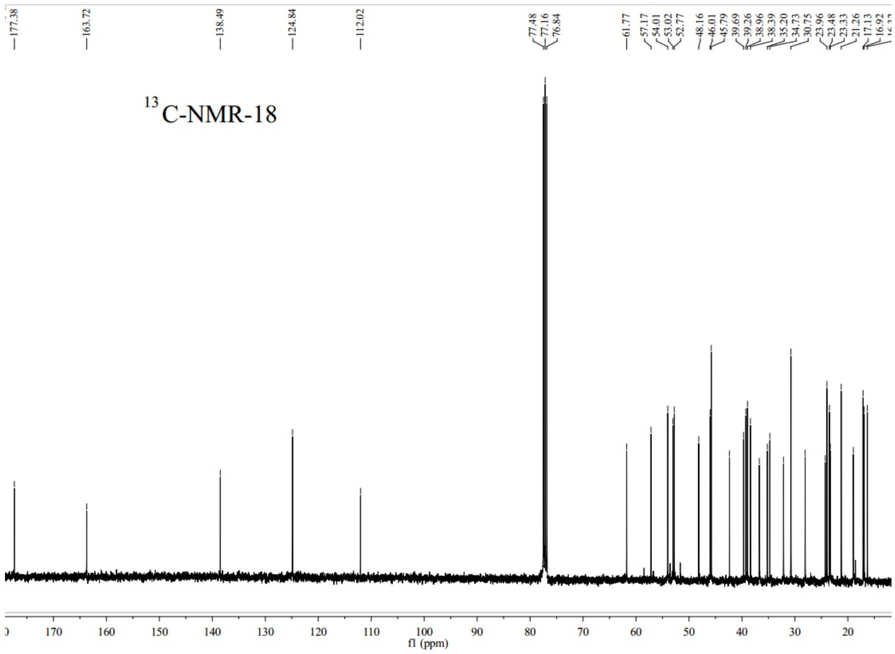

Figure S23. ^{13}C NMR spectra of **18**

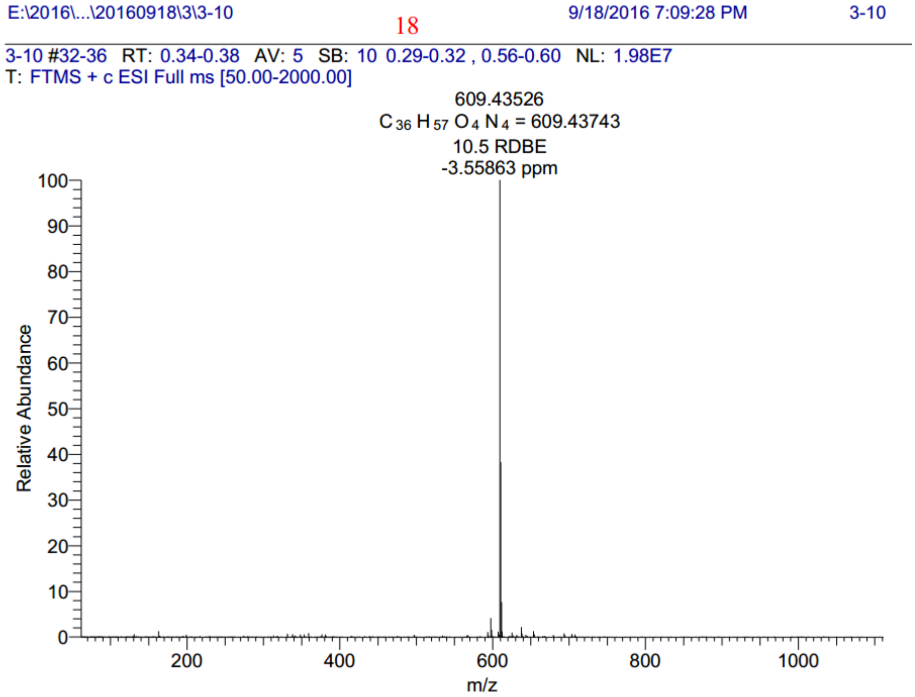

Figure S24. HRMS spectra of **18**
